# *Ret* and *Etv4* Promote Directed Movements of Progenitor Cells during Renal Branching Morphogenesis

**DOI:** 10.1371/journal.pbio.1002382

**Published:** 2016-02-19

**Authors:** Paul Riccio, Cristina Cebrian, Hui Zong, Simon Hippenmeyer, Frank Costantini

**Affiliations:** 1 Department of Genetics and Development, Columbia University, New York, New York, United States of America; 2 Department of Microbiology, Immunology, and Cancer Biology, University of Virginia School of Medicine, Charlottesville, Virginia, United States of America; 3 Center for Brain Immunology and Glia, University of Virginia School of Medicine, Charlottesville, Virginia, United States of America; 4 Developmental Neurobiology, IST Austria (Institute of Science and Technology Austria), Klosterneuburg, Austria; University of Cambridge, UNITED KINGDOM

## Abstract

Branching morphogenesis of the epithelial ureteric bud forms the renal collecting duct system and is critical for normal nephron number, while low nephron number is implicated in hypertension and renal disease. Ureteric bud growth and branching requires GDNF signaling from the surrounding mesenchyme to cells at the ureteric bud tips, via the Ret receptor tyrosine kinase and coreceptor Gfrα1; Ret signaling up-regulates transcription factors Etv4 and Etv5, which are also critical for branching. Despite extensive knowledge of the genetic control of these events, it is not understood, at the cellular level, how renal branching morphogenesis is achieved or how Ret signaling influences epithelial cell behaviors to promote this process. Analysis of chimeric embryos previously suggested a role for Ret signaling in promoting cell rearrangements in the nephric duct, but this method was unsuited to study individual cell behaviors during ureteric bud branching. Here, we use Mosaic Analysis with Double Markers (MADM), combined with organ culture and time-lapse imaging, to trace the movements and divisions of individual ureteric bud tip cells. We first examine wild-type clones and then *Ret* or *Etv4* mutant/wild-type clones in which the mutant and wild-type sister cells are differentially and heritably marked by green and red fluorescent proteins. We find that, in normal kidneys, most individual tip cells behave as self-renewing progenitors, some of whose progeny remain at the tips while others populate the growing UB trunks. In *Ret* or *Etv4* MADM clones, the wild-type cells generated at a UB tip are much more likely to remain at, or move to, the new tips during branching and elongation, while their *Ret−/− or Etv4−/−* sister cells tend to lag behind and contribute only to the trunks. By tracking successive mitoses in a cell lineage, we find that *Ret* signaling has little effect on proliferation, in contrast to its effects on cell movement. Our results show that Ret/Etv4 signaling promotes directed cell movements in the ureteric bud tips, and suggest a model in which these cell movements mediate branching morphogenesis.

## Abstract 2

The cellular mechanisms of branching morphogenesis in the developing kidney are unclear. Mosaic analysis shows that branching involves epithelial cell movements controlled by the Ret receptor tyrosine kinase and the transcription factor Etv4.

## Author Summary

During kidney development, the growth and repeated branching of an epithelial tube, the ureteric bud, generates the tree-like collecting duct system. In humans, defects in these processes cause congenital abnormalities of the kidney and urinary tract. While many of the genes that control these events are known (such as the signaling receptor *Ret* and the transcription factor *Etv4*), the cellular mechanisms underlying ureteric bud branching remain unclear. By time-lapse microscopy of mouse fetal kidneys developing in culture, in which individual ureteric bud cells are fluorescently labeled, we show that most cells at the tips behave as progenitors, some of whose daughters remain at the tips while others populate the tubular uretic bud trunks. We then use Mosaic Analysis with Double Markers, a genetic method that generates pairs of mutant and wild type cells, each labeled with a different fluorescent protein. We compare the behaviors of the mutant or wild-type ureteric bud cells by time-lapse microscopy of organ development in culture and by examining their numbers and spatial distribution after kidney development in vivo. The results reveal that *Ret* and *Etv4* promote epithelial cell movements within the branching ureteric bud tips and suggest a model in which such cell movements mediate branching.

## Introduction

Kidney development begins with the outgrowth of the primary ureteric bud (UB) from the nephric duct into the adjacent metanephric mesenchyme. The UB then bifurcates repeatedly during fetal development, generating ~10^4^ terminal branches (in mouse) that connect to nephrons, the renal filtering units [1–3]. Normal UB growth and branching is essential for the development of a full complement of nephrons, and low nephron number is thought to be a risk factor for development of hypertension and chronic kidney disease in humans [4].

A key stimulus of UB branching morphogenesis is the secreted protein GDNF, which signals via the receptor tyrosine kinase Ret and coreceptor Gfrα-1 [5,6]. *Gdnf* is expressed initially by the metanephric mesenchyme cells, and then by the nephron progenitor cells (also known as “cap mesenchyme”) that surround each UB tip throughout kidney development [7–11]. *Ret* is expressed by cells throughout the terminal UB “tips”, but not by cells in the “trunks” (the tubular portions behind the tips that elongate, narrow, and differentiate to form the collecting ducts) [12]; *Gfra1* is expressed in both cap mesenchyme and UB tip cells [13–15]. In mice lacking any of these genes, the UB usually fails to form, causing renal agenesis, or else branches minimally, causing severe renal hypoplasia [5,16]. Mutations in these genes are also sometimes associated with renal agenesis or other congenital defects of the kidney or urinary tract in humans [6,17]. While the requirement for GDNF, and for several other signals (including fibroblast growth factors [FGFs], Wnts, and bone morphogenetic proteins [BMPs]), to achieve the normal extent and pattern of renal branching (and, indirectly, nephron number) is well established [2,17,18], the specific cellular behaviors controlled by these signals, and how these behaviors contribute to the branching morphogenesis of the UB epithelium, remain unclear.

Branching morphogenesis underlies the development of many organs and involves the transition of a simple epithelial tube, or a mass of cells, into a complex, branched structure. There are several cellular mechanisms by which an epithelial tube might bifurcate, including changes in cell shape, oriented cell division, clefting by extracellular matrix filaments, or cell movements, among others [19–22]. In some organs, there is evidence that a particular mechanism contributes to branching—e.g., apical cell constriction in lung budding [23] or clefting by extracellular matrix filaments in salivary gland branching [24]—but for the most part, the cellular basis of branching remains unknown. Some insight into the role of Ret signaling in cell behaviors in the nephric duct, during formation of the primary UB, was obtained by the analysis of chimeric embryos. In these studies, a fraction of nephric duct cells were mutant for *Ret*, for *Etv4* and *Etv5* (two closely related E26 transformation-specific [ETS] transcription factors that act downstream of Ret) [25], or for *Spry1* (a negative regulator of Ret signaling) [26], and the remainder were wild-type [27,28]. The results suggested a competitive process in which Ret signaling stimulates a subset of nephric duct cells, experiencing higher levels of Ret signaling, to move to the site of bud evagination and form the tip of the primary UB.

These findings raised the question whether *Ret*- and *Etv4/Etv5*-dependent cell movements also orchestrate the more complex patterns of branching morphogenesis that occur during kidney development. Several types of cell movement have been observed in the UB and other branching epithelia [29–33], but how they are controlled, and their roles in branching morphogenesis, are largely obscure. The analysis of chimeric embryos was not suited to address this question, because cells lacking *Ret*, or *Etv4* and *Etv5*, were excluded from the UB branches at the outset of kidney development [27,28].

Therefore, we employed Mosaic Analysis with Double Markers (MADM) [34] to induce rare clones, containing mutant and wild-type UB cells, at various stages of renal organogenesis. Starting with *Ret+/−* embryos (whose kidneys are phenotypically close to normal) [3,35], Cre-mediated interchromosomal recombination between MADM sequence elements on chromosome 6 (MADM6) generates rare cells that contain a *Ret* wild-type allele linked to one fluorescent reporter (e.g., Tomato) and a *Ret* null allele linked to a different reporter (e.g., GFP). When such cells divide, a GFP+, *Ret−/−* daughter and a Tomato+, *Ret+/+* daughter are generated in some cases, while in other cases a double-labeled daughter and an unlabeled daughter (both remaining *Ret*+/−) are produced [34,36] (S1 Fig). The behaviors of these labeled cells and their progeny were assessed either by time-lapse microscopy of organ cultures or by examining kidneys that had developed in vivo. Similar clonal analyses using mouse strains with MADM cassettes located on chromosome 11 (MADM11) [37] allowed us also to evaluate the role of *Etv4* in UB tip cell behavior. This novel application of clonal analysis, coupled with time-lapse imaging, provided new insights into the role of the *Ret*/*Etv4* pathway in UB cell behaviors during branching morphogenesis.

## Results

### Ureteric Bud Tip Cells Are Progenitors of Tip and Trunk Cells

Previous data suggested that the UB tips contain a progenitor cell population that, upon cell division, generates new tip cells (forming the exponentially increasing number of UB tips during kidney development) as well as cells that will populate the UB trunks [38,39]. However, the fates of individual UB tip cells and their descendants have not been examined. For this purpose, we followed the behaviors of rare, fluorescently labeled clones that arose during UB branching in renal organ cultures. These labeled clones had the same genotype as the rest of the kidney, except for fluorescent reporter genes (Materials and Methods and **S1 Fig**). The kidneys were explanted between E11.5 and E13.0, cultured for 2–3 d, and time-lapse microscopy was used to visualize the appearance, divisions, and movements of the individual labeled cells and their clonally-related descendants.

**Fig 1:**
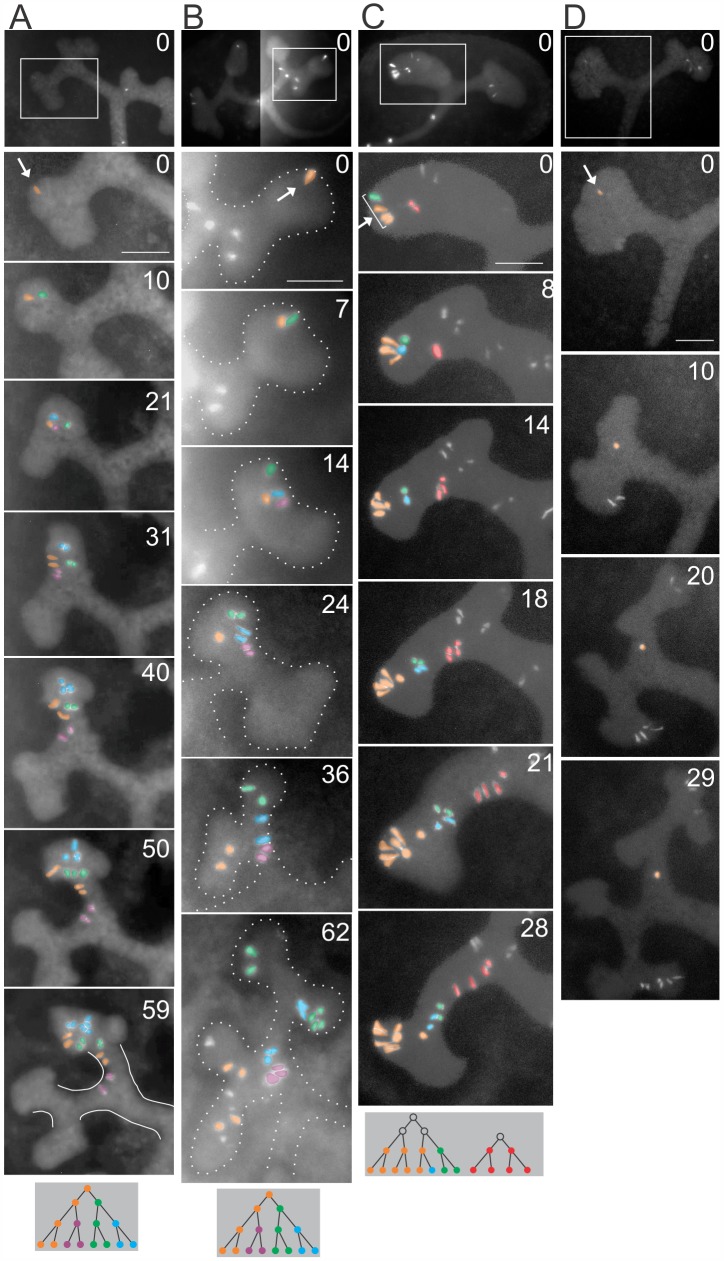
Tracking individual clones initiating in a UB tip. E11.5 or E12.0 kidneys were cultured and time-lapse imaged by fluorescence microscopy. The top image in each column shows the entire UB at the time the labeled clone was first detected (= 0 h). The founder cell(s) (arrow) and its daughters are pseudocolored, as indicated by diagrams at the bottom. **A**–**C**, three clones that arise at a tip and distribute cells between new tips and trunks. **A**, as the tip extends and starts to branch (0–21 h), the clone remains in a cluster near one tip. As the next-generation branches extend (31–40 h), the clone distributes along the tip-trunk axis. By 50–59 h, six cells are in the trunk (outlined in white), and the remainder in the tip. **B**, as the tip branches, one daughter (green) moves past its orange sister to the upper tip (7–14 h), while the others remain in the central cleft. The green subclone remains near the tips during two more branching generations. The cleft narrows to form a trunk (14–24 h), where the purple subclone remains during further branching. The blue subclone is divided between this trunk and a new tip (62 h). Meanwhile, the orange subclone moves far from its siblings, traversing the tip (14–36 h) and ending in the opposite branches. **C**, at 0 h, the clone consists of four cells at a tip (three orange and one green). As the tip extends (8 h), the green cell and one daughter of an orange cell (now colored blue) lag behind the others, and their four daughters end up in the trunk. Meanwhile, the other (orange) cells in this clone remain at the tip, except for one that starts to leave the tip at 18 h. The red clone starts in the middle of the tip and ends entirely in the trunk (28 h). As the trunk elongates and narrows (18–28 h), the red and green/blue cell clusters change from a staggered to a linear arrangement, an apparent example of convergent extension [30]. **D**, an example of a labeled cell that arises in a tip but ends in the trunk, never dividing. Scale bars, 100 μm. See also **S1 Movie**.

We analyzed 62 labeled clones, each of which arose from an individual labeled cell in the UB tips (in 34 kidneys), following these clones during the first ~5–6 generations of branching, which readily occur in organ cultures (**Fig 1, S1 Movie**). By the end of the cultures, in 39% of these clones, the daughter cells were distributed among the tips and trunks (e.g., **Fig 1A–1C, S1 Movie**), in 16% of clones, all cells remained in the tips for the duration of the culture, while 45% of the clones had formed only trunk cells (e.g., **Fig 1C and 1D, S1 Movie**). Thus, most individual tip cells retain the potential to “self-renew” (i.e., to produce some daughters that remain in the progenitor population at the tip), while others have lost this ability and contribute only to the trunks.

These time-lapse studies also allowed us to measure directly the individual cell cycle times by noting when each successive division occurred in a cell lineage (**S2 Fig**). Previously, based on kinetics of EdU incorporation, the average cell cycle time in UB tips in vivo was estimated to be 11.7 h at E13.25, slowing to 23.5 h at E17.25 [39]. Those data suggested a unimodal distribution of cell cycle lengths in the UB tips, unlike the cap mesenchyme, which contained a faster-cycling and a slower-cycling subset of cells [39]. We also observed a unimodal distribution of cell cycle times in the tips, with a mean of 14.5 h and a range of 8–30 h. This is only slightly slower than the in vivo estimate [39], a difference that may be due to the effects of in vitro culture. Cell cycles in the trunks were generally longer, averaging 22.4 h and ranging from 11 h to >40 h. This supports the conclusion from BrdU incorporation studies [40] or phosphohistone H3 staining [32] that cells in the tips proliferate more rapidly than those in the trunks.

We confirmed that UB tip cells, collectively, are the progenitor population for most, if not all, collecting duct cells, by using a tip-specific, inducible Cre recombinase allele (*Ret* ^*CreERT2*^) to permanently express yellow fluorescent protein (using the *Rosa26R-YFP* reporter allele) in many tip cells and examining the kidneys after development in vivo (**S3 Fig**) or during in vitro culture (**S2 Movie**). Together, these data indicate that a subset of tip cells remain at the tips and maintain the progenitor population, while others lag behind and form the trunks.

### *Ret* Influences Ureteric Bud Cell Movement and Cell Position in Organ Cultures

**Fig 2:**
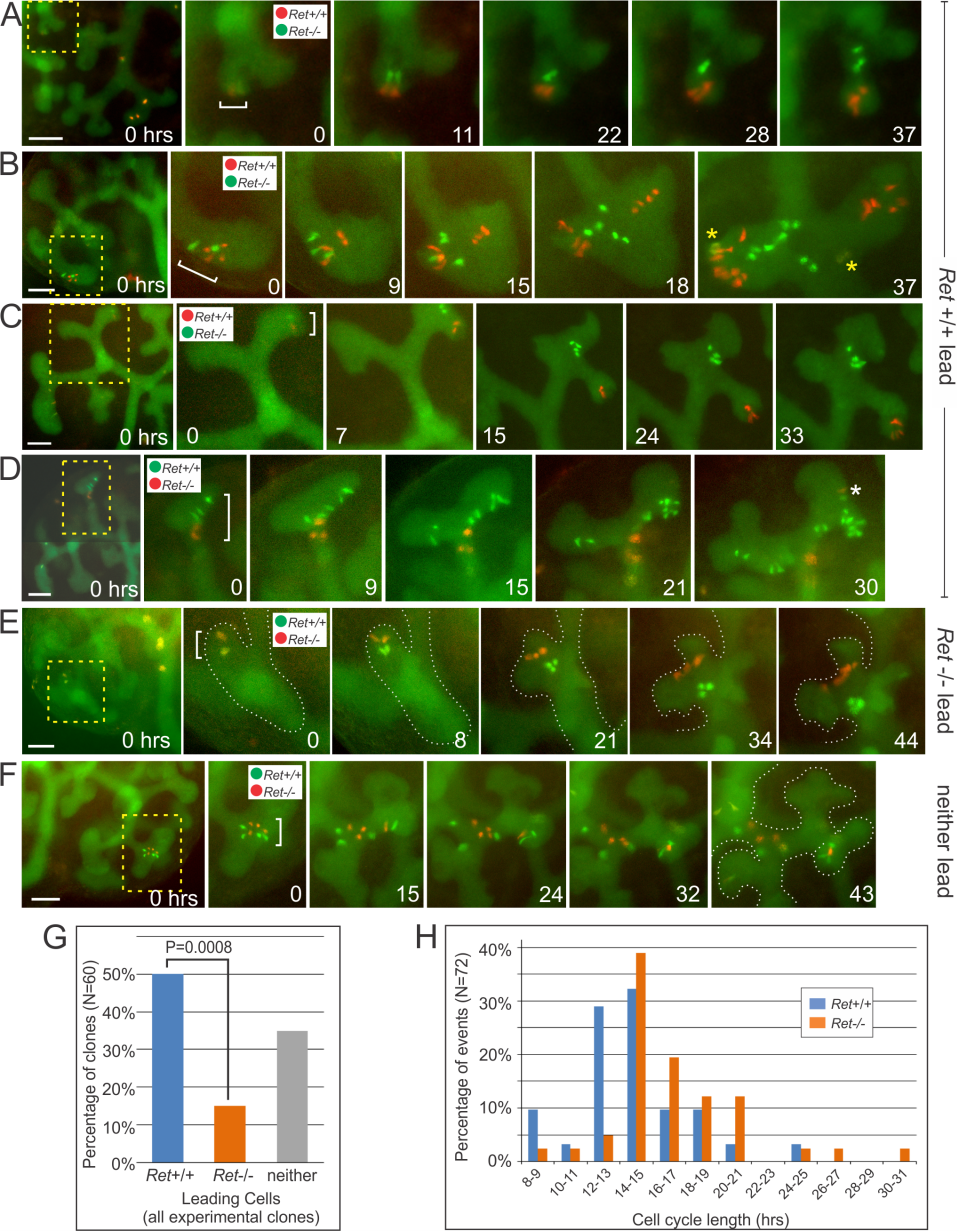
Time-lapse analysis of *Ret*-MADM clones in cultured kidneys. Twenty-four E12.0- E13.0 kidneys were cultured and imaged every 20–30 min. The entire UB was labeled weakly with GFP by *Hoxb7/CreGFP* [41], whose Cre activity induced MADM recombination in a rare, random subset of UB cells. This generated pairs of red/green sister cells, one *Ret*+/+ and the other *Ret*−/− (in 14 kidneys *Ret*+/+ cells were red, and in 10 kidneys *Ret*+/+ cells were green, depending on whether the *MADM6-TG* or *MADM6-GT* allele was in *cis* to the *Ret* null allele—see **S1 Fig**). **A–F,** development of six *Ret*-MADM clones. 0 h is when the clone, marked by a bracket, was first detected. Each image sequence contains a low-mag. view at 0 h (scale bar, 100 μm), followed by images of the clone at selected times. **A–D,** four clones in which *Ret*+/+ cells ended closer to the UB tip(s) than their *Ret*-/- sisters. **E**, a clone in which *Ret*−/− cells ended closer to tips. **F**, a clone in which neither *Ret+/+* nor *Ret−/−* cells were clearly closer to the tips. Insets in **A**–**F** indicate the genotypes of red and green cells. Yellow asterisks in B indicate newly arising “yellow” clones (*Ret*+/−); the white asterisk in D marks an independently arising red/green clone. **G**, percentages of *Ret*-MADM clones in which *Ret*+/+ or *Ret*−/− cells ended closer to the UB tips, or in which neither was clearly closer. **H**, cell cycle lengths for individual *Ret*+/+ or *Ret*−/− cells (measured while they remained within, or very close to, the UB tips). Time-lapse sequences are shown in **S3 Movie**. Data for Fig 2G and 2H available from the Dryad Digital Repository: http://dx.doi.org/10.5061/dryad.pk16b [42].

What determines which cells will remain at the tips as the ureteric bud grows and branches, and which will be left behind to form the trunk epithelium? We tested the hypothesis that Ret signaling influences this decision, using MADM6 recombination driven by a UB-specific Cre transgene (*Hoxb7/CreGFP*). Starting with a *Ret*+/− kidney, this genetic method (**S1 Fig**) generates rare clones consisting, initially, of a *Ret*+/+ cell and a *Ret*−/− sister cell, which are differentially labeled with Tomato (red) or GFP (green; and readily detected despite weak GFP signal throughout the UB from *Hoxb7/CreGFP*). Kidneys were explanted between E12.0 and E13.0, cultured for ~36–48 h, and all of the red and green cells in each clone were followed in time-lapse movies (**Fig 2**, **S3 Movie**). In 50% of the red/green clones (*n* = 60) that arose in the UB tips, most or all of the *Ret+/+* cells moved ahead of the *Ret−/−* cells as the UB extended and branched, and ended up closer to the new tips than the *Ret−/−* cells (**Fig 2A–2D and 2G**). In only 15% of the clones did *Ret−/−* cells end up closer to the tips than the *Ret*+/+ cells (**Fig 2E and 2G**), while in 35% there was no clear “leader” (**Fig 2F and 2G**). Thus, *Ret*+/+ cells were >3 times more likely to remain, or move, closer to the UB tips than their *Ret*−/− sisters. Depending on the initial arrangement of *Ret* and *MADM6* alleles (**S1 Fig**), the *Ret*+/+ cells could be either red or green; a similar difference in the behaviors of wild-type versus mutant cells was observed in kidneys where the *Ret*+/+ cells were red (*Ret*+/+ lead in 19 clones and trail in 4) or green (*Ret*+/+ lead in 11 clones and trail in 5), showing that there was no inherent bias in the behavior of Tomato+ versus GFP+ cells. In many cases (e.g., in **Fig 2B**, the upper-right red cell at 0 h, and its daughters), *Ret+/+* cells moved a considerable distance, from the center or base of a tip into a newly emerging tip, suggesting that the *Ret*+/+ cells have a competitive advantage in the ability to populate the new tips, not only over their *Ret*−/− sisters but also over the *Ret+/−* cells that comprise most of the UB epithelium.

One type of cell rearrangement in the ureteric bud tips occurs at mitosis (termed “mitosis-associated cell dispersal”, MACD), when one daughter immediately moves, via the lumen, to a site approximately 1–3 cells distant, while the other daughter remains at the site of origin [32]. Both *Ret*+/+ and *Ret*−/− cells underwent MACD following most mitoses (**S4 Fig**). However, this was not the only mechanism by which *Ret*+/+ cells moved relative to *Ret*−/− cells, as many changes in the relative positions of wild-type versus mutant cells also occurred in between cell divisions (**S4 Fig**).

We examined the effects of *Ret* on UB cell proliferation by measuring the final number of red and green cells per clone (each clone begins as one red and one green cell), and also by noting the time intervals between successive mitoses (as shown for wild-type cells in **S2 Fig**). Both *Ret+/+* and *Ret−/−* UB cells had a mode of cell cycle lengths at 14–15 h, but in *Ret*–/–cells, the distribution was shifted towards longer cell cycles (**Fig 2H**), and the average was slightly longer than in *Ret*+/+ cells (16.7 ± 3.9 h versus 14.4 ± 3.4 h; *p* < 0.02, *t* test). Similarly, the average MADM clone ended with 3.8 ± 3.3 *Ret*+/+ cells versus 3.1 ± 2.1 *Ret*−/− cells (*p* < 0.01, *t* test). However, the effect of *Ret* on cell proliferation appeared likely to be a secondary consequence of its effect on cell position: among the 30 clones where *Ret*+/+ cells ended closer to the tips, the average ratio of *Ret+/+* to *Ret−/−* cells was 1.36, whereas among the nine clones where *Ret*−/− cells ended closer to the tips, the ratio was 0.74, and among the 21 clones where there was no clear difference in position, the average ratio was 1.14. Thus, in MADM clones, *Ret*+/+ cells apparently proliferate more than their *Ret*−/− sister cells because they usually remain closer to the UB tips, and probably not because of an inherent effect of *Ret* on cell proliferation.

The death of a GFP+ or Tomato+ cell, when it occurred, was visible in the time-lapse movies: the cell fragmented, then disappeared (**S4D Fig**). However, cell death was rare—5 *Ret*+/+ and 1 *Ret*−/− cell deaths were observed among ~250 *Ret*+/+ and ~200 *Ret*−/− cells—and thus did not contribute significantly to the numbers of *Ret*+/+ versus *Ret*−/− cells. Even this low level of cell death is likely a culture artifact, as UB cell death is virtually undetectable in kidneys developing in vivo [41,43].

### *Ret* Influences Ureteric Bud Cell Position in E17.5 Kidneys

**Fig 3:**
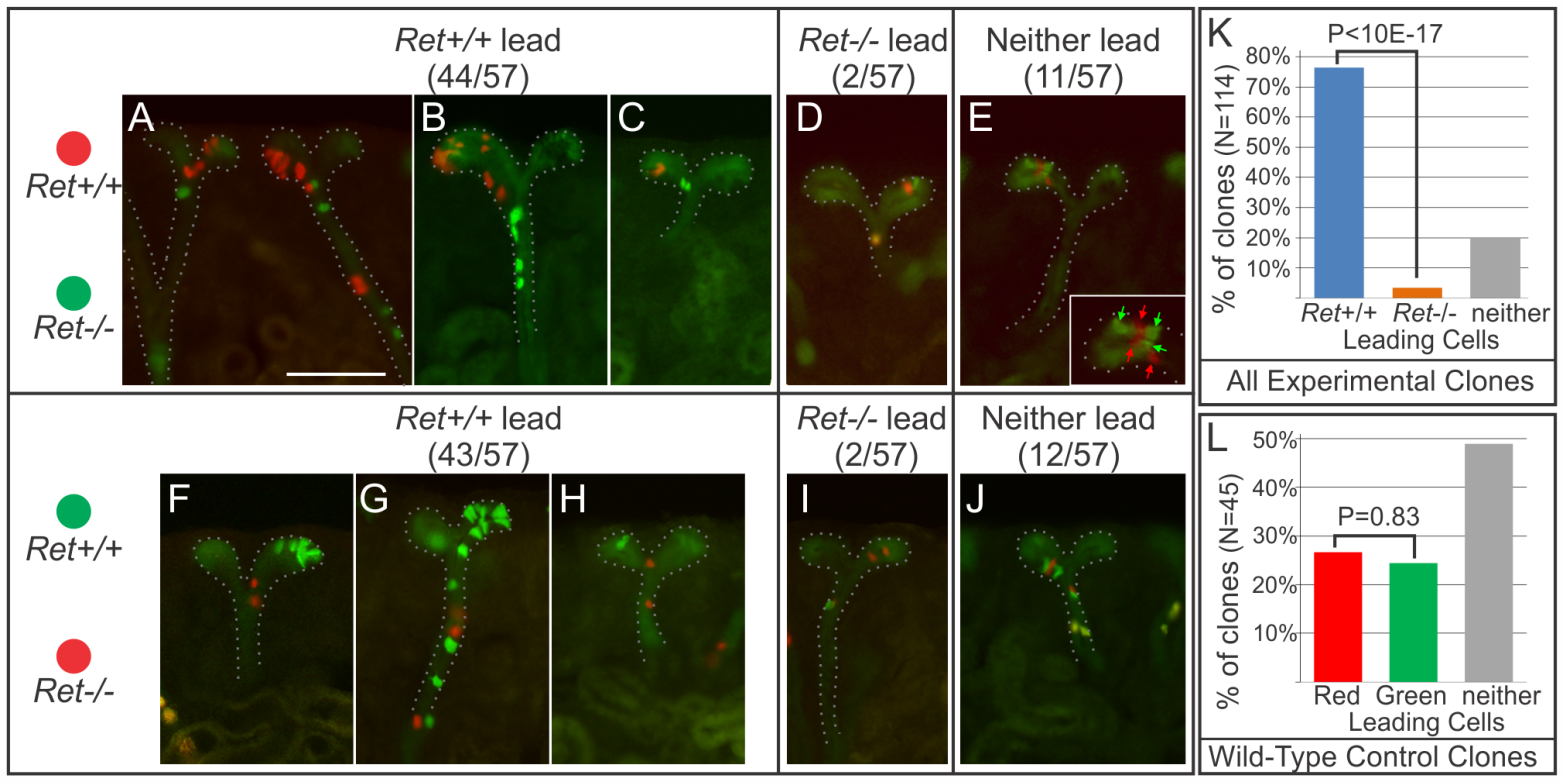
Analysis of *Ret*-MADM clones in E17.5 kidneys. MADM recombination was induced in a rare, random subset of UB cells by *Hoxb7/Cre-GFP*. Ten kidneys were vibratome-sectioned (50 μm) and red/green clones that included cells in a UB tip were scored (because such clones must have arisen from a UB tip cell, whereas clones located entirely in the interior of the kidney might have arisen in a UB trunk cell, where *Ret* is not expressed). **A–E,** examples of MADM clones from kidneys in which *Ret*+/+ cells were red and *Ret*−/− cells green. Inset in E shows an enlargement of the clone, with the positions of the red and green cells indicated by arrows. **F–H,** examples of clones from kidneys in which *Ret*+/+ cells were green and *Ret*−/− cells red. **K**, overall percentages of clones in which the *Ret*+/+ cells were closer to the UB tips, the *Ret*−/− cells were closer to the tips, or neither was clearly closer. **L**, similar analysis of red/green clones in three control MADM kidneys, in which both red and green cells were wild-type. Scale bar 100 μm. Data for Fig 3K and 3L available from the Dryad Digital Repository: http://dx.doi.org/10.5061/dryad.pk16b [42].

To ask whether *Ret* has similar effects on UB tip cell behavior in vivo, we retrospectively analyzed 114 *Ret*-MADM6 clones in E17.5 kidneys (**Fig 3**); as *Hoxb7/CreGFP* is active in the UB throughout kidney development, these clones may have arisen by recombination at any prior stage. In 76.3% of the clones, the *Ret*+/+ cells were clearly closer to the UB tips than the *Ret*−/− cells (**Fig 3A–3C**, **3F–3H and 3K**), while the *Ret*−/− cells were closer to the tips in only 3.5% of clones (**Fig 3D**, **3I and 3K**), and there was no clear difference in 20.2% of clones (**Fig 3E, 3J and 3K**). The results were similar in experiments where the *Ret*+/+ cells were Tomato+, and those where the *Ret*+/+ cells were GFP+ (**Fig 3**). In control E17.5 MADM6 kidneys, in which every cell was wild-type, there was no difference in the average position of GFP+ versus Tomato+ cells (**Fig 3L**).

Among the E17.5 *Ret*-MADM6 clones, there was a more pronounced difference in the number of *Ret*+/+ versus *Ret*−/− cells than seen in the cultured E12.0–13.0 kidneys. The average clone contained 8.7± 29 *Ret*+/+ cells and 2.1±1.4 *Ret*−/− cells. However, these averages were skewed by a few very large clones with up to several hundred *Ret*+/+ cells, and many fewer *Ret*−/− cells (as MADM recombination driven by *Hoxb7/CreGFP* can occur at any stage of kidney development, these large clones at E17.5 presumably resulted from early recombination events): the median number of cells per clone (a statistic that is insensitive to a few very large clones) was 3 *Ret*+/+ and *2 Ret*−/− cells. The numbers of clones containing different ratios of *Ret*+/+ to *Ret*−/− cells is shown in a histogram in **S5 Fig**. In the control, wild-type MADM clones, the average number of Tomato+ cells (3.9 ± 3.5) versus GFP+ cells (4.5 ± 5.9) per clone was not significantly different (*p* = 0.53, *t* test). Thus, during kidney development, the *Ret*+/+ cells in an average MADM clone proliferate more than the *Ret*−/− cells, but, as discussed below, this is likely a secondary effect of their locations within the UB.

### *Etv4* Has Effects Similar to *Ret* on UB Tip Cell Rearrangements

**Fig 4:**
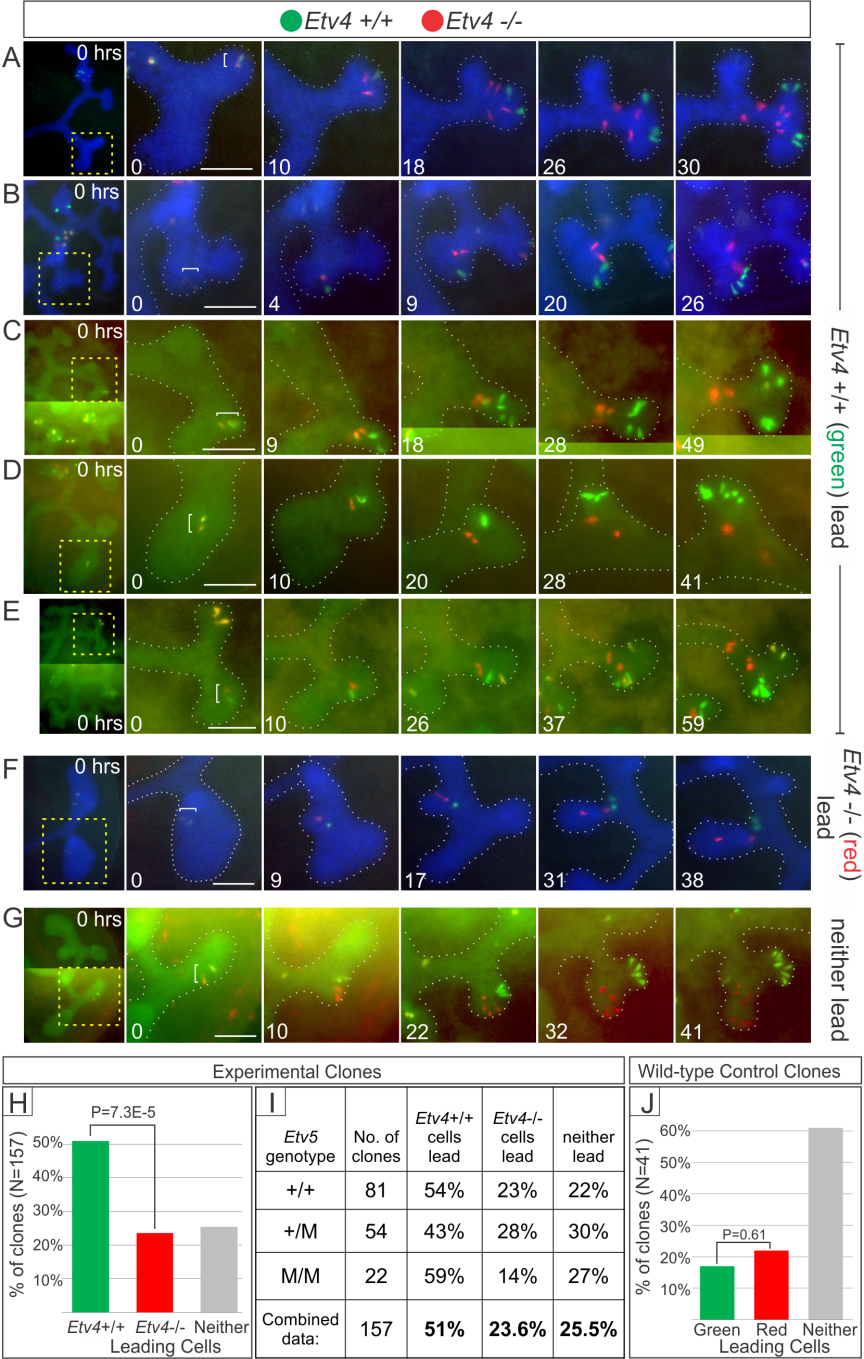
Time-lapse analysis of *Etv4*-MADM clones in cultured kidneys. Forty-four E11.5−E12.5 kidneys, in which rare MADM recombination was induced by *Hoxb7/Cre* [46], were cultured and imaged every 30 or 60 min. In all cases, green cells were *Etv4*+/+ and red cells were *Etv4*−/−; yellow clones were *Etv4*+/−, like the starting kidney. **A–G** show the development of seven *Etv4*-MADM clones. **A–E**, five clones in which *Etv4*+/+ cells ended closer to the UB tip(s) than their *Etv4*−/− sisters. **F**, one clone in which *Etv4*−/− cells ended closer to the tip than the *Etv4*+/+ cells. **G**, one clone in which *Etv4+/+* and *Etv4−/−* cells were equally close to two UB tips. The kidneys in **A**–**C** and **G** were *Etv5*+/+, those in **D** and **F** were *Etv5*+/M, and the kidney in **E** was *Etv5*-M/M. The kidneys also carried R26R-CFP [47], expressed throughout the UB (after recombination by *Hoxb7/Cre*), and visible in the blue channel (**A**, **B**, **F**) or by spillover to the green channel (**C**–**E**, **G**). **H**, percentage of *Etv4*-MADM clones (including all *Etv5* genotypes) in which *Etv4*+/+ cells ended closer to the UB tips, or *Etv4*−/− cells ended closer to the tips, or neither was clearly closer. **I**, comparison of the *Etv4*-MADM results in kidneys of different *Etv5* genotypes. **J**, analysis of MADM clones in control kidneys, in which both red and green cells were wild type for *Etv4*. Scale bars, 100 μm. Time-lapse sequences are shown in **S4 Movie**. Data for Fig 4H–4J available from the Dryad Digital Repository: http://dx.doi.org/10.5061/dryad.pk16b [42].

**Fig 5:**
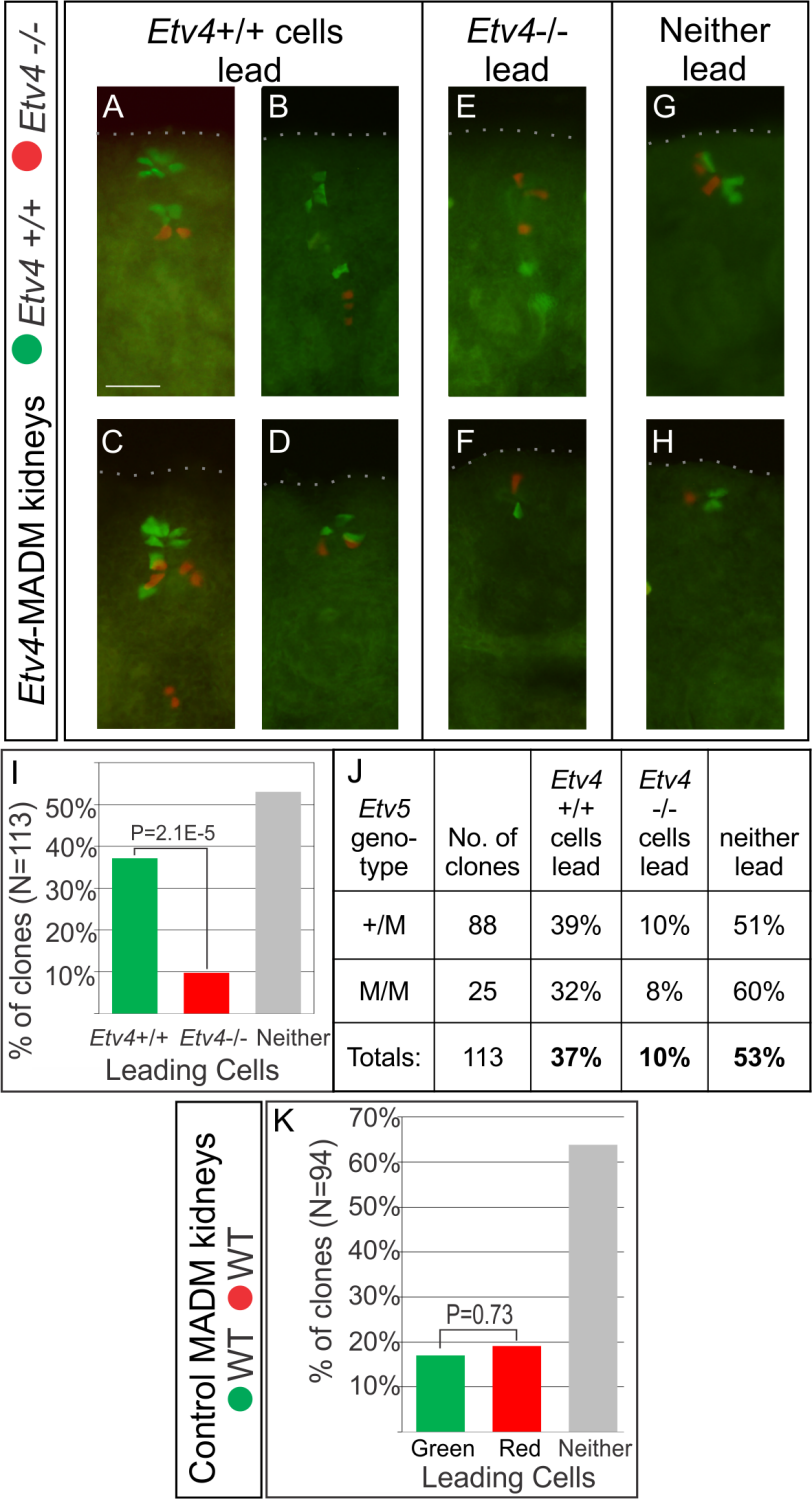
Analysis of *Etv4*-MADM clones in E16.5 kidneys. MADM recombination was induced in rare cells throughout the UB by *Hoxb7/Cre* [46]. Six kidneys were vibratome-sectioned (50 μm); all red/green clones that included cells ≤50 μm from the edge of the kidney (dotted line), and thus close to the tips, were scored (as the outline of the UB epithelium was not always visible). In all clones, green cells were *Etv4*+/+ and red cells were *Etv4*−/−. **A–D**, examples of clones in which the *Etv4*+/+ cells were closer to the UB tips; **E–F**, examples in which *Etv4*−/− cells were closer to the tips; **G–H**, examples in which neither was clearly closer to the tips. Scale bar in **A**, 50 μm. The *Etv5* genotypes were +/M (**A**–**C**, **E**, **G**, **H**) or M/M (**D**, **F**). **I**, percentage of all *Etv4*-MADM clones in which *Etv4*+/+ or *Etv4*−/− cells were closer to the UB tips, or neither was clearly closer. **J**, comparison of *Etv4*-MADM results for kidneys in which the *Etv5* genotype was +/M or M/M (there were no *Etv5*+/+ kidneys). **K**, analysis of clones in control MADM kidneys, in which both red and green cells were wild type for *Etv4*. Data for Fig 5I–5K available from the Dryad Digital Repository: http://dx.doi.org/10.5061/dryad.pk16b [42].

The transcription factors *Etv4* and *Etv5* act downstream of *Ret* and are jointly required for normal UB branching [25] and for nephric duct cell rearrangements during formation of the primary UB [28]. Using MADM strains for chromosome 11 [37], where *Etv4* is located, in combination with *Hoxb7/Cre*, we performed clonal analyses to examine the effect of an *Etv4* null mutation [44] on the behavior of UB tip cells. Because the renal defects of *Etv4−/−* kidneys are exacerbated by *Etv5* mutations (*Etv4*−/− mice have a very low frequency of renal hypoplasia or agenesis, while *Etv4*−/− mice also carrying one or two mutant *Etv5* alleles have a much higher frequency of these defects) [25], some embryos also carried 1 or 2 copies of a hypomorphic *Etv5* allele (*Etv5* ^*tm1Kmm*^, abbreviated *Etv5* ^*M*^) [25,45]. The results for *Etv4*-MADM11 (**Figs 4** and **5**, **S4 Movie**) were similar to those observed for *Ret*-MADM6. Among 157 red/green clones analyzed in cultured kidneys (including all three *Etv5* genotypes), the *Etv4*+/+ cells ended closer to the UB tips in 51.0% of clones (e.g., **Fig 4A–4E**), the *Etv4−/−* cells in only 23.6% (e.g., **Fig 4F**), and there was no clear difference in 25.5% (e.g., **Fig 4G**). The difference in behavior of *Etv4*+/+ versus *Etv4*−/− cells was highly significant (**Fig 4H**), and this disparity was observed for all *Etv5* genotypes (**Fig 4I**). In control MADM11 cultures, where all cells were wild type, there was no significant difference between the behavior of red and green cells (**Fig 4J**).

The average cell cycle lengths for *Etv4*+/+ versus *Etv4*−/− UB tip cells were indistinguishable (15.1 ± 3.0 versus 15.4 ± 3.5 h, respectively). Another measure of cell proliferation, the final number of cells per clone, showed a small but significant difference: 3.4 ± 3.3 *Etv4*+/+ cells versus 3.1 ± 2.4 *Etv4*−/− cells per clone (*p* = 0.04, *t* test). Thus, loss of *Etv4* has only a minor effect on cell proliferation; the somewhat greater effect of *Ret* than *Etv4* on proliferation probably reflects its similarly stronger influence on cell position (compare **Figs 2G** and 4 **H**).

The positions of the *Etv4* mutant versus wild-type cells in 113 additional *Etv4*-MADM11 clones were analyzed retrospectively in vibratome sections of E16.5 kidneys (**Fig 5A–5H)**. In 37% of clones, the green *Etv4*+/+ cells were closer to the UB tips than the red *Etv4*−/− cells, while the *Etv4*−/− cells were closer in only 10% (in 53%, neither the *Etv4*+/+ nor *Etv4*−/− cells were unambiguously closer) (**Fig 5I**). As in the *Etv4*-MADM11 time-lapse studies, an “advantage” of *Etv4*+/+ over *Etv4*−/− cells was observed in both *Etv5* ^*M/+*^ and *Etv5* ^*M/M*^ genetic backgrounds (**Fig 5J**). In control E16.5 MADM11 kidneys, in which both red and green cells were wild-type, there was no difference in the average position of red versus green cells (**Fig 5K**). Thus, the loss of *Etv4*, like *Ret*, strongly reduces the likelihood that a UB tip cell will remain near the new tips as the UB extends and branches.

### Wild Type Ureteric Bud Tip Cells Exhibit Heterogeneous Levels of Ret Signaling, and Undergo Extensive Spatial Rearrangements

**Fig 6:**
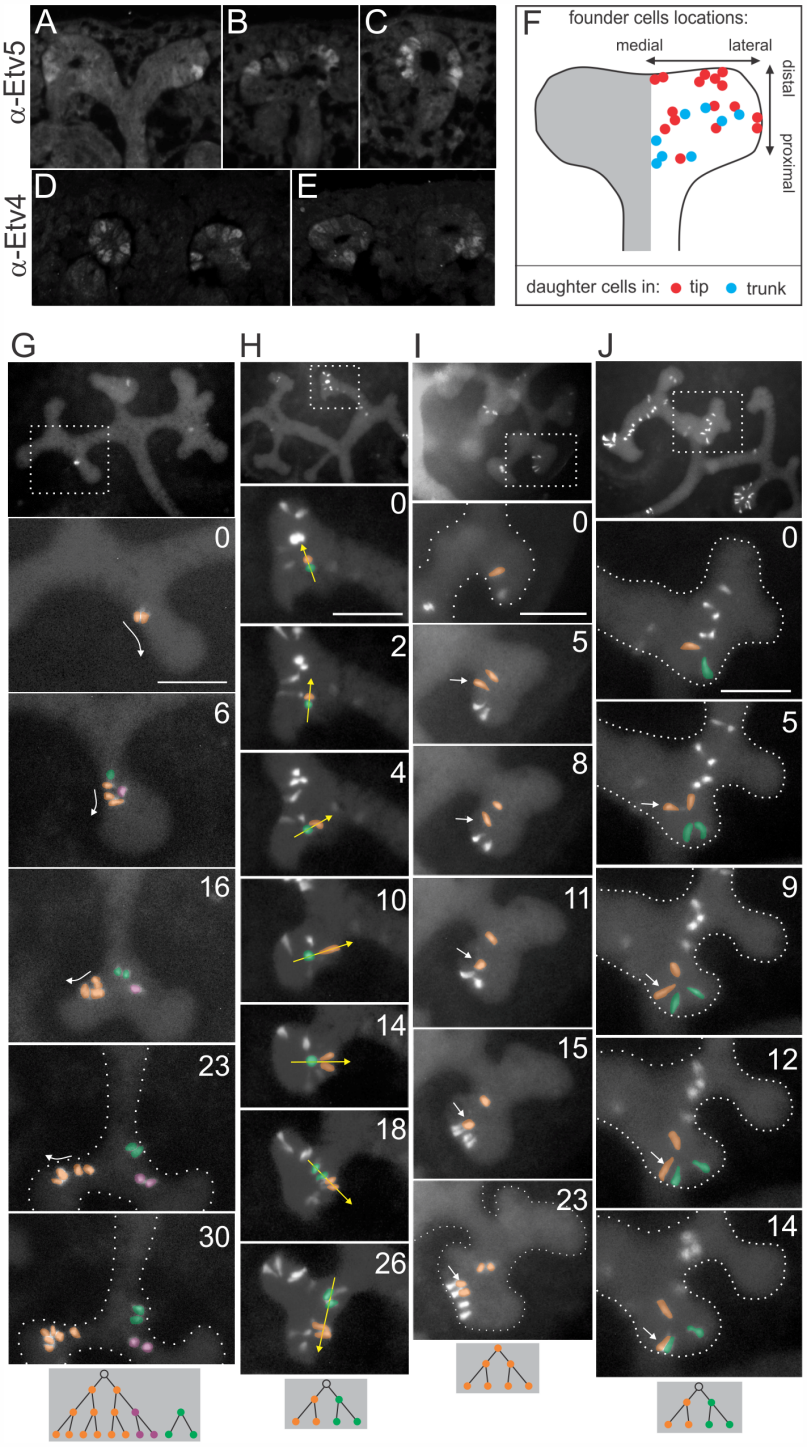
Heterogeneous expression of Etv4 and Etv5, and cellular rearrangements, among wild-type ureteric bud tip cells. **A**–**E**, Heterogeneous expression of Etv4 and Etv5. Wild type E14.5 kidneys were stained with antibodies against Etv5 (**A**–**C**) or Etv4 (**D**–**E**). Both proteins are strongly expressed only in an interspersed subset of ureteric bud tip cells. **F**, Fate-mapping of tip cells contributing to tip or trunk. The starting locations of labeled wild-type clones (individually tracked as shown in Fig 1) are plotted according to their positions on an idealized UB tip (see Materials and Methods); tip cells giving rise to daughters that stayed at the tip for the duration of the cultures are colored red, while tip cells whose daughters were found only in trunks are colored blue. **G**–**J**, Cell movements during wild-type UB branching. **G,** one clone (two cells at 0 h, colored orange) originates at the base of a tip. Both cells divide by 6 h (one of the four daughters is then colored purple). The three orange cells move forward (arrows) as branching occurs, and by 30 h occupy one of the new tips. The purple cell moves away and ends in the opposite branch. The green cell (an independent clone) appears at ~6 h, very close to the orange clone, but does not undergo the same forward movements and remains behind in the trunk. **H**, two sister cells (orange and green) rotate around each other by >180° as the branch extends and branches again. While the green subclone is initially closer to the tip, it recedes to the trunk (14–26 h) while the orange subclone moves ahead. **I**, after the orange cell divides, one daughter (arrow) moves towards a tip, while the other daughter remains at the base of the tip. **J**, after the orange cell divides, one daughter (arrow) moves to the tip. The UB epithelium is demarcated by dotted white lines when not clearly visible. Scale bars, 100 μM. The diagrams at the bottom show the lineage relationships among the pseudocolored cells. Time-lapse sequences are shown in **S5 Movie**. Data for Fig 6F available from the Dryad Digital Repository: http://dx.doi.org/10.5061/dryad.pk16b [42].

In the MADM clonal analyses, the *Ret* or *Etv4* genotype of a UB tip cell strongly influenced its ability to stay close to the new tips during UB branching. How do these observations pertain to the development of a normal kidney, in which all cells are wild-type for *Ret* and *Etv4*? In contrast to the expression of *Ret* mRNA, which appears similar among all UB tips cells [e.g., Fig 5 in 48], we found that the levels of Etv4 and Etv5 proteins, whose expression is up-regulated by Ret signaling [25], are extremely heterogeneous among wild-type UB tip cells (**Fig 6A–6E**). Vsnl1, another protein whose expression in the UB tip requires Ret signaling, was also shown to have a mosaic pattern of expression [49]. Thus, even wild-type UB tip cells exhibit strong variation in levels of Ret signaling markers. As some transcription factors are expressed in pulsatile patterns [reviewed in 50], which cannot be discerned by antibody staining, it remains to be determined whether the variable levels of Etv4 and Etv5 proteins represent stable changes or pulsatile fluctuations.

To ask if the future contribution of a UB tip cell to the new tips versus the trunks could be predicted from its location within the branching UB tip, we measured the starting locations of labeled (wild-type) clones, which had been tracked in time-lapse movies (as in Fig 1). The location of the founder cell for each clone along the proximal–distal and medial–lateral axes was plotted on an idealized UB tip (**Fig 6F**). Only cells with daughters that stayed at the tips (red symbols) were found in the most distal region, but throughout most of the UB tip, they were located similarly to cells that generated only future trunk cells (blue symbols). This suggests that clones starting out in similar locations can end up in different regions of the branching UB.

This analysis predicted that neighboring cells within an individual UB tip may sometimes move in different directions, a prediction that was confirmed by the time-lapse analysis of labeled wild-type clones (**Fig 6G–6J**, **S5 Movie**). For example, in **Fig 6G**, three cells (pseudocolored orange, 6 h) move from the base of a tip towards one of the newly forming tips; meanwhile, a sibling cell (purple) and a nearby independent clone (green) move far away to populate the trunk of the opposite branch. In **Fig 6H**, the cells colored orange and green (two sisters from a recent division) orbit around each other by >180°, and the initially-trailing orange subclone ends up closer to the tip. In **Fig 6I** and **6J**, one of two sister cells (arrow) moves forward towards the tip, while the other sister remains at the base of the tip. Thus, even in kidneys where all cells have the same genotype, some UB tip cells undergo long-range movements comparable to those seen in kidneys that are genetically mosaic for *Ret* or *Etv4*. Together, these data suggest a model in which natural cell-to-cell variation in the level of Ret signaling influences the behavior and fate of UB tip cells during normal branching morphogenesis.

## Discussion

**Fig 7:**
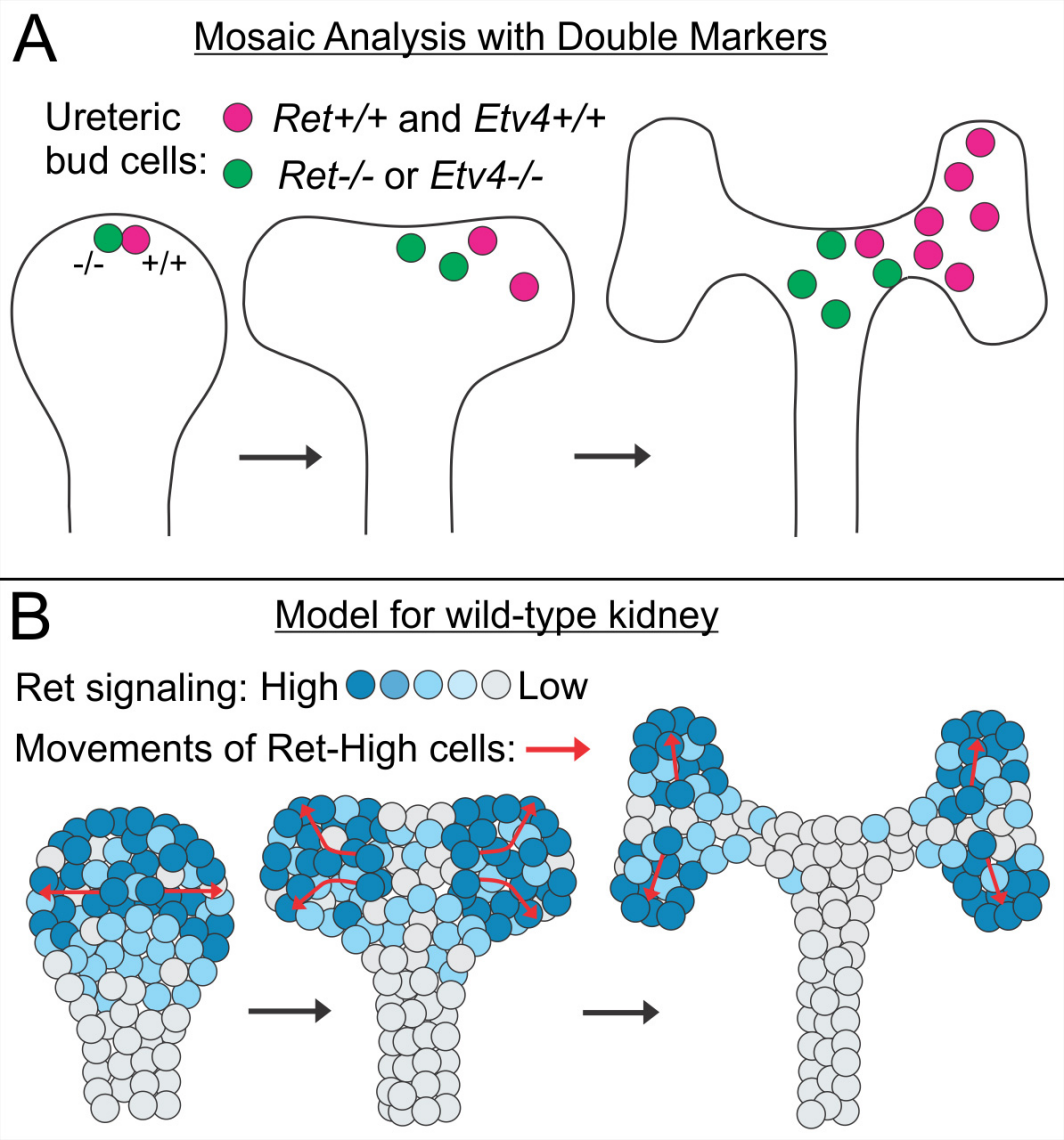
Behavior of *Ret*+/+ versus *Ret*−/− and *Etv4*+/+ versus *Etv4*−/− ureteric bud tip cells in MADM clones, and model for the effect of Ret signaling on tip cell behavior in wild-type kidneys. **A,** In *Ret* or *Etv4* MADM clones, the wild-type cells (red) tend to remain closer to the new ureteric bud tips than their *Ret*−/− or *Etv4*−/− sister cells (green) as the ureteric bud branches. **B**, In wild-type kidneys, Ret signaling is lost in a fraction of tip cells (Fig 6A–6E); we suggest that the cells retaining high levels of Ret signaling move towards the sites where new tips are forming (red arrows), thus contributing to branching, while the cells with reduced Ret signaling lag behind and form the new trunks. During normal development, this appears to be primarily a one-way process. The de novo formation of tips from trunks has been observed in cultured kidneys, during rare lateral branching events [51], and can be induced by the juxtaposition of isolated UB trunks with isolated metanephric mesenchyme [56]. The molecular mechanisms of such de novo tip formation, and whether it occurs during kidney development in vivo, remain unclear.

Morphogenesis of the renal collecting system involves repeated cycles of ureteric bud branching, during which the tips elongate and then bifurcate (or sometimes trifurcate) to form new branches [3,51,52]. New cells are added primarily by cell division in the tips [32,38,40], although cells in the trunks (and, later, in the elongating collecting ducts) also continue to divide at a lower rate [32,53] (**S2 Fig**). Here, we first showed, by time-lapse analysis of individual clones in cultured wild-type kidneys, that many individual UB tip cells give rise to daughters that remain at the growing tips, during one or more branch generations, and to other daughters that are left behind to form the elongating trunks. This, together with other types of cell lineage tracking [38] (**S3 Fig**), establishes that the tips constitute the main progenitor population for UB growth, as has been observed in several other branching epithelial structures [22,54,55]. We then investigated the genetic mechanisms that determine which cells in a UB tip will remain at the tips during subsequent elongation and branching. Based on previous studies of cell behaviors during primary ureteric budding from the nephric duct [27,28], we hypothesized that Ret signaling, acting in part via the transcription factors *Etv4* and *Etv5*, plays an important role in this process. We therefore used MADM to generate fluorescently labeled clones, each initiating as a wild-type cell and a sister cell lacking *Ret* or *Etv4*, in an otherwise heterozygous organ; we tracked these clones during branching morphogenesis in organ cultures, or analyzed their spatial distributions in fixed kidneys. Such tip cell clones became progressively dispersed as the kidneys grew, and we observed a strong propensity for *Ret*+/+ or *Etv4*+/+ cells to remain closer to the UB tips than their *Ret*−/− or *Etv4*−/− sister cells (**Fig 7A**). This reveals that Ret signaling, at least in part via its effect on *Etv4* expression [25], can strongly influence a cell’s ability to remain at the tips of the expanding ureteric tree.

The effect of loss of *Ret* or *Etv4* on tip cells was not absolute, as in a small proportion of MADM clones, the mutant UB cells remained closer to the tips than the wild-type cells, and in other clones, there was no clear difference in their positions; however, the tendency of wild-type cells to take the “lead” was highly significant, in both time-lapse analyses of cultured, early-stage kidneys and (to an even greater extent) in retrospective analyses of E16.5 or E17.5 fetal kidneys. Thus, while the *Ret* or *Etv4* genotype is not the sole determinant of tip cell fate in such genetically mosaic kidneys, it is an important one. One explanation for the instances in which wild-type cells failed to show an advantage is that Ret signaling is normally heterogeneous among UB tip cells, even in fully wild-type kidneys (as revealed by varying levels of diphospho-Erk, or *Etv4* or *Etv5* expression, from cell to cell). Thus, even some of the *Ret*+/+ or *Etv4*+/+ tip cells in MADM clones presumably lose Ret signaling activity and are thus fated to leave the tip and form trunk. Another potential reason is that several other growth factors and receptors [2,18], including FGF10 and FGFR2 [57], promote UB branching in cooperation with GDNF and Ret [48] and may influence UB cell behavior in similar ways; perhaps some *Ret*−/− cells are “rescued,” at least temporarily, by FGF signaling.

Loss of *Etv4* alone has only minor effects on kidney development, while the defects are greatly exacerbated by *Etv5* mutations, indicating that these two transcription factors act somewhat redundantly [25]. For this reason, we performed *Etv4*-MADM in different *Etv5* genetic backgrounds; yet loss of *Etv4* strongly influenced UB cell behavior even in an *Etv5*+/+ background. Thus, cells lacking *Etv4* are at a competitive disadvantage in the ability to stay at the UB tip, even if they retain *Etv5* (this experiment did not test if *Etv5* also affects cell position, since all the cells in each kidney had the same *Etv5* genotype). We also found that *Ret*+/+ cells not only remained closer to the tips than *Ret*−/− cells, but in many cases they moved towards, and concentrated at, the extreme UB tips, revealing an advantage over the *Ret*+/− cells that constitute most of the UB epithelium in *Ret*-MADM6 kidneys. Thus, relatively minor differences in the level of Ret signaling may confer an advantage in a competition among UB cells to move toward the sites where new tips are forming.

The role of GDNF/Ret signaling in UB tip cell proliferation had been unclear, as one previous study indicated that exogenous GDNF promoted the proliferation of UB tip cells [58], while another showed no such effect [15]. Here, we could directly assess the cell-autonomous effect of loss of Ret signaling on UB tip cell proliferation, by observing the cell cycle lengths of the *Ret*+/+ versus *Ret*−/− cells in MADM clones, in time-lapse movies. Our results showed that *Ret−*/− tip cells divided only slightly less often than their *Ret*+/+ sister cells. Because the *Ret*−/− cells typically lagged behind their *Ret*+/+ sisters, and since cell proliferation in wild-type kidneys becomes slower as UB cells leave the tips and enter the trunks, it is likely that the modest effect of *Ret* genotype on tip cell proliferation is a secondary consequence of its effect on cell movement (and thus cell position). In *Etv4*-MADM clones, we also observed only a small difference between the proliferation of *Etv4*+/+ and *Etv4*−/− UB cells. Thus, the strongest effect of the *Ret* and *Etv4* genotypes was an influence on the ability of tip cells to remain near the tips during kidney growth. Apparently, in wild type kidneys, the higher rate of proliferation of tip versus trunk cells is mainly due to the mitogenic effects of other factors expressed by the cap mesenchyme cells that surround the tips, such as FGFs [41], and not primarily to GDNF.

Why do *Ret* and *Etv4* wild-type cells tend to remain closer to the branching UB tips than their mutant sister cells? We propose that many (but not all) cells in the UB tips move in the direction of growth of the newly forming tips, and that Ret signaling, via Etv4 and Etv5, promotes these cell movements (**Fig 7B**). Presumably, Etv4 and Etv5 induce the expression of effector genes that participate in cell movement; candidates include the chemokine receptor *Cxcr4* and the matrix metalloprotease *Mmp14* [25]. How these cell movements are guided remains unclear; they could be directed by diffusible signals from the cap mesenchyme, or from other tip cells, or perhaps by other positional cues such as gradients of cell adhesion [59]. An obvious candidate for a chemoattractive signal is GNDF, as *Ret*-expressing cultured cells can migrate towards a source of GDNF [60], and GDNF appears to act as a chemoattractant for enteric neural crest cells [61,62]. The cap mesenchyme cells expressing GDNF are broadly distributed around the entire UB tip, and it is thus unlikely that the expression pattern of GDNF generates gradients that could direct cell movements towards the specific sites of new tip formation; however, it remains possible that GDNF gradients are shaped by secondary mechanisms, such as binding to extracellular matrix components [63–66], or other mechanisms of ligand sequestration or redistribution [67–69]. Alternatively, Ret signaling via Etv4/Etv5 could up-regulate the expression of another receptor(s) for a different signal(s) that provides the positional information for tip cell movements; or it might promote general cell motility per se (although this was not apparent from examination of mutant versus wild-type UB cells in the time lapse movies).

While our observations on the role of *Ret* and *Etv4* in cell behaviors were made in genetically mosaic kidneys, we observed extensive cell movements in the normal UB epithelium, similar to those seen in the genetically mosaic kidneys. Furthermore, in wild type UB tips, the level of Ret signaling activity appeared to be extremely heterogeneous from cell to cell. This suggests a model in which Ret signaling is somehow repressed in a subset of UB tip cells but maintained in others, generating UB tips that are “mosaic” in Ret signaling, although genetically wild-type (**Fig 7B**). The mechanism by which such heterogeneity is generated remains unclear. According to this model, the tip cells with reduced Ret signaling are fated to become trunk cells and fail to move toward the newly forming tips, while those cells retaining high levels of Ret signaling preferentially move tipward. Since GDNF/Ret signaling maintains *Ret* gene expression through a positive feedback loop [25,58], the *Ret*-expressing tip cells with reduced signaling activity will eventually lose *Ret* expression as well. Our results support the hypothesis that *Ret*-dependent cell movements are part of the basic mechanism by which the UB tip epithelium changes its shape, thus forming new branches [60,70]. Thus, the abnormalities in UB branching caused by mutations affecting the Ret pathway, in mice and humans, are likely to be caused by defects in epithelial cell movements.

How do UB cells move with respect to their sisters and other neighboring cells? Several mechanisms of UB cell movement have been described, including MACD, short-range cell intercalation, and rosette rearrangement. MACD causes the rearrangement of most dividing cells within the UB tips, due to the jumping of one daughter to a noncontiguous location [32]. But these are relatively short-range movements (typically 1–3 cell diameters), they are apparently unaffected by lack of *Ret* or *Etv4*, and they occur at mitosis, while we observed extensive tip cell movements in between cell divisions. Rosette resolution and cell intercalation have been documented in elongating mouse-collecting ducts and frog pronephric duct [30,31], but these are also relatively short-range forms of cell movement; in contrast, we observed long-range movements of labeled cells in the UB tips, both wild type and genetically mosaic, which are unlikely to be explained by these mechanisms. It thus appears that UB epithelial cells are capable of extensive migration within the epithelium during branching morphogenesis.

Mutant clonal analysis is an important approach to study gene function during development and has been used widely in lower organisms such as the fly, but infrequently in mammalian systems. MADM [34,71], like MARCM in Drosophila [72], is a particularly powerful method, as it generates pairs of mutant/wild type sister cells that are differentially labeled, and whose behaviors can be directly visualized and compared. While MADM has been used to study the distribution of mutant/wild type clones in neural development and cancer [73–75], it has not been previously combined with time-lapse analysis to study cell behaviors during organogenesis. While the genes that can be studied using this approach are currently limited to those on chromosomes for which MADM alleles have been generated, currently chromosomes 6, 7, 11, and 12 [34,36,37,76], the generation of additional MADM strains will extend the number of chromosomes amenable to this powerful method of clonal analysis.

## Materials and Methods

### Animals

All experiments were approved by the Columbia University Institutional Animal Care and Use Committee (Protocol AAAH4954). All mice were on a mixed genetic background. The following alleles were used:

MADM6-TG (Mouse Genome Informatics: Gt(ROSA)26Sortm7(ACTB-EGFP*)Luo) [36], MADM6-GT (Mouse Genome Informatics: Gt(ROSA)26Sortm6(ACTB-EGFP*,-tdTomato)Luo) [36], MADM11-TG (Mouse Genome Informatics: Tg(ACTB-tdTomato,-EGFP)11Luo) [37], MADM11-GT (Mouse Genome Informatics: Tg(ACTB-EGFP,-tdTomato)11Luo) [37], *Ret* ^*CreERT2*^ (Mouse Genome Informatics: Rettm2(cre/ERT2)Ddg) [77] (Note: This was used as a *Ret*-null allele, not for its tamoxifen-inducible Cre activity; it has no Cre activity in the absence of tamoxifen, and even when we attempted to use this allele with tamoxifen to drive recombination, no MADM recombinant clones were observed), Hoxb7/Cre (Mouse Genome Informatics: Tg(Hoxb7-cre)13Amc) [46], Hoxb7/CreGFP (Mouse Genome Informatics: Tg(Hoxb7-cre)5526Cmb) [41], Rosa26R-CFP (Mouse Genome Informatics: Gt(ROSA)26Sortm2(ECFP)Cos) [47], Rosa26R-tdTomato (Mouse Genome Informatics:Gt(ROSA)26Sortm14(CAG-tdTomato)Hze/J) [78], Rosa26R-YFP (Mouse Genome Informatics: Gt(ROSA)26Sortm1(EYFP)Cos) [47], Etv4-NLZ (Mouse Genome Informatics: Etv4tm1Arbr) [44], Etv5-M (Mouse Genome Informatics: Etv5tm1Kmm) [45].

### Clonal Analysis of Normal UB Tip Cells

Kidneys were cultured and imaged by epifluorescence microscopy, as described [79]. MADM recombination was induced by *Hoxb7/Cre* [46] or *Hoxb7/CreGFP* [41], expressed throughout the UB, but only clones arising in the tips were analyzed. These included “yellow” clones from *Ret*-MADM6 or *Etv4*-MADM11 (which retain the same heterozygous genotype as the rest of the kidney; **S1 Fig**) and wild-type red/green clones in control MADM kidneys. The movies were converted to grayscale and cells pseudocolored using CorelDraw (**Figs 1** and **6G–6J**) or marked with colored dots using ImageJ (**S1 and S3–S5 Movies**).

To plot the starting locations of labeled wild-type clones (**Fig 6F**), we selected clones in UB tips that were at a stage of branching at which (width at the tip)/(width at the “neck”) was ≥ 2, but in which the two nascent tips had not yet begun to branch again; for example, in **Fig 7** this would include the tip in the middle but not the tip on the left (too narrow) nor the one on the right (nascent tips already branching again). As the shapes of the initial T-shaped UB tips at E11.5 were very heterogeneous, only clones arising in later branching generations were used; the latter were also excluded if the tip shape was too irregular to accurately measure the cell position. The position of each founder cell was measured along the proximal–distal and medial–lateral axes of the tip, and a symbol was drawn at the corresponding position on the idealized UB tip shown in **Fig 6F**. Only the right side of the idealized UB tip was used to plot cell positions, as UB tips are normally bilaterally symmetric.

### Analysis of *Ret*-MADM6 and *Etv4*-MADM11 Clones

A *Ret* null allele [77] was placed in *cis* to MADM6-TG and MADM6-GT alleles [34] by meiotic recombination. *Ret*-MADM6-GT/+ mice (both sexes) were mated to MADM6-TG/+ or TG/TG mice carrying *Hoxb7/CreGFP*, and *Ret*-MADM6-TG/+ mice (both sexes) were mated to MADM6-GT/+ or GT/GT mice carrying *Hoxb7/CreGFP*. E12.0, 12.5 or 13.0 kidneys were cultured and time-lapse imaged [79]. All red/green clones that arose near a UB tip were scored at the end of the culture (or at the latest time all cells remained in-frame and in-focus). Clones in which (a) the average position of red (or green) cells was closer to a tip, and (b) the cell closest to a tip was red (or green), were scored as red (or green) “leading”; clones that did not meet both criteria were scored “neither leading”. In vibratome sections of E16.5–17.5 kidneys, all red/green clones (defined as clusters of cells well-separated from other clusters) that included at least one cell in a tip were scored by the same criteria as for time-lapse movies. Clones limited to more interior regions of the kidney were not scored, as they may have arisen in a trunk cell, where *Ret* and *Etv4* are not expressed.

For MADM11 [37], *Etv4*−/− or +/−;MADM11-GT/GT; *Etv5*+/M females were mated to *Hoxb7/Cre;* MADM11-TG/TG; *Etv5*+/M; *R26R*-CFP/CFP males. Embryos were genotyped to identify those inheriting a mutant *Etv4* allele from the mother (by necessity, in *cis* to MADM11-GT), and to determine the *Etv5* genotype. Red/green recombinant clones were analyzed as described for *Ret*-MADM6.

### Antibody Staining

For anti-Etv4 and anti-Etv5 staining, thin frozen sections were processed for immunofluorescence as previously described [80]. Rabbit anti-Etv4 primary antibody, a gift from Dr. Thomas Jessell, was diluted 1:50 in TSP (0.1% Triton X-100, 0.05% [w/v] Saponin in PBS). Rabbit anti-Etv5 (Proteintech 13011-1-AP) was diluted 1:100 in TSP. Fluorescently conjugated secondary antibodies (Jackson ImmunoResearch) were diluted 1:400 in TSP. Samples were imaged on a Zeiss Axioscope epifluorescence microscope.

For anti-calbindin staining, fetal kidneys were fixed overnight at 4˚C in 4% paraformaldehyde in PBS. After washing 3x in PBS, kidneys were mounted in 3% agarose, vibratome-sectioned at a thickness of 50–60 μm, and sections were postfixed in methanol for 15 min. Vibratome sections were blocked with 2% donkey serum in PBT, and primary and secondary antibody incubations were performed in PBT. Sections were incubated with goat anti-calbindin (1:400, Santa Cruz) and rabbit anti-GFP (1:500, Invitrogen) overnight at 4˚C. Following at least three washes with PBS, Cy5-conjugated donkey anti-goat and Cy2-conjugated donkey anti-rabbit secondary antibodies (1: 500) were applied overnight at 4˚C (Jackson ImmunoResearch). Sections were rinsed several times in PBS and mounted on slides with Fluoro-Gel (Electron Microscopy Sciences).

## Supporting Information

S1 Fig Generation of labeled cells using MADM. The key in the upper right explains the symbols used. In the starting cell (top diagram), shown in G1 phase, one chromosome 6 homolog carries the *MADM-GT* allele [34,36] at the *Rosa26* locus in *cis* to a *Ret*-null allele (−), and the other chromosome 6 homolog carries the *MADM-TG* allele at the *Rosa26* locus in *cis* to a wild-type *Ret* allele (+). Thus, the cell is heterozygous for *Ret*. Neither the *MADM-GT* nor *MADM-TG* allele expresses a functional fluorescent protein [34,36]. If Cre-mediated recombination occurs during G1 phase (or during G0, or in postmitotic cells), it generates a functional Tomato (red fluorescent protein) gene and a functional GFP gene in the same diploid cell, so the cell is double-labeled and appears yellow. It also remains heterozygous for *Ret*. If Cre-mediated recombination occurs during G2, after DNA replication, the recombination products include a functional Tomato gene in *cis* to a *Ret* wild-type allele, and a functional GFP gene in cis to a *Ret*-null allele (shown on chromatids 2 and 3, respectively), as well as two non-recombined, non-functional MADM alleles (on chromatids 1 and 4). At mitosis, the four chromatids segregate in either of two patterns, X-segregation or Z-segregation. The former yields a *Ret*−/−, GFP-expressing cell and a *Ret* +/+, Tomato-expressing cell. The latter yields an unlabeled cell and a double-labeled (yellow) cell, both of which remain *Ret*+/−. Subsequent cell divisions preserve the genotype and fluorescent protein expression of the initial recombinant cells. The strategy for *Etv4*-MADM was similar, except we used MADM alleles on chromosome 11 [37], where *Etv4* is located. Diagram modified from [34].
(TIF)

S2 Fig Direct measurement of ureteric bud cell cycle times in tip vs. trunk. Cell cycle times were measured by noting when each labeled cell divided. **A**, images of a cultured kidney (the same one shown in **Fig 1A**), in which the identity of each cell in a labeled clone is marked. **B**, the complete lineage of the clone shown in **A**, from 0 to 59 h. The *y*-axis indicates the time of cell division (also shown in black numbers between each pair of sister cells). Red numbers indicate the time between two successive mitoses, i.e., cell cycle time. Green circles indicate cells that divided in the tip, and blue circles indicate cells that divided in the trunk. **C**, distribution of cell cycle times in the tip and trunk, based on 150 intermitotic intervals in UB tips and 33 in UB trunks. For trunk cells, red bars indicate the time between two successive mitoses, and orange bars indicate the time from a cell division until the end of the movie (i.e., the minimum cell cycle time). Cells were classified as “tip” or “trunk” based on their location at the end of the cell cycle (i.e., the second of the two successive mitoses) or at the end of the movie, whichever came first. Data available from the Dryad Digital Repository: http://dx.doi.org/10.5061/dryad.pk16b [42].
(TIF)

S3 Fig Fate-mapping *Ret*-expressing ureteric bud tip cells in vivo. **A**, genetic strategy for fate-mapping *Ret*-expressing cells. To follow the fate of UB tip cells at different stages of kidney development in vivo, we used a transgenic line, *Ret* ^*CreERT2*^, in which a tamoxifen-inducible form of Cre was targeted to the *Ret* locus and is thus expressed in the pattern of the *Ret* gene [77]. *Ret* ^*CreERT2*^ mice were crossed with *Rosa26R* ^*YFP*^ mice, in which YFP is permanently expressed from the *Rosa26* locus only after a floxed “stop” sequence is removed by Cre-mediated recombination [47,81]. **B**, timing of tamoxifen injection and analysis. Pregnant females were injected with a single 2 mg dose of tamoxifen at E11.5, E13.5, E15.5, or E16.5. This induces Cre activity starting about 6–8 hours later, and continuing for about 24 hours [82,83]. The embryos were all dissected at E17.5, the kidneys were vibratome-sectioned (50 μm), and YFP fluorescence was photographed. As expected, given the tip-restricted expression of *Ret* in the UB throughout kidney development [12] (GUDMAP.org), when recombination was induced at E16.5, YFP was expressed at E17.5 only in cells close to the UB tips, at the edge of the kidney (**F**). In contrast, when recombination was induced at E11.5 (when *Ret* is expressed broadly in the first two UB branches), YFP+ cells were found at E17.5 all along the collecting ducts, from the papilla to the distal tips (**C**). When recombination was induced at E13.5, YFP+ cells were found at E17.5 throughout most of the collecting ducts, except for the papillary region (**D**); and when it was induced at E15.5, YFP+ cells were found at E17.5 from the cortical CDs to the tips, but not in the medullary or papillary regions (**E**). As expected, all cells labeled by *Ret* ^*CreERT2*^ remained within the collecting ducts, as confirmed by costaining for YFP and calbindin, a collecting duct marker (**G-I**). Scale bars: 500 μm.
(TIF)

S4 Fig Both *Ret*+/+ and *Ret*−/− cells undergo MACD and also move during interphase; cell death is rare. **A–C**, three examples of MACD and cell rearrangement in *Ret*-MADM clones (**B** shows the same clone as Fig 3B, and **C** shows the same clone as Fig 3F, but additional time points are shown here to highlight MACD and other cell movements). **A**, the green *Ret*−/− cell (green arrow) starts closer to the left UB tip than the red *Ret*+/+ cell (red arrow), but by 4.7 h they have exchanged positions. The *Ret*−/− cell divides between 3 and 3.3 h (white arrows) and the *Ret*+/+ cell divides between 5.3 and 5.7 h, both displaying MACD (seen as rounding of the parental mitotic cell, and in the next frame as two non-contiguous daughter cells). Between 5.7 and 8.3 h, both *Ret*+/+ cells continue to move closer to the left UB tip than the two *Ret*−/− cells. The insets show the separate red and green channels when a red and green cell overlap, between 3.3 and 5.7 h. **B**, a green *Ret*−/− cell (white arrow) undergoes MACD between 2.7 and 3.0 h. Between 6.7 and 10 h, two red *Ret*+/+ cells (red arrows) move past a green *Ret*−/− cell (white arrow) towards the site where a new tip is forming. Between 12 and 14.3 hrs, three red *Ret*+/+ cells (red arrow) move left towards the site of tip outgrowth, passing a green *Ret*−/− cell (yellow arrow). **C**, in a clone at the center of a T-shaped branching tip, a green *Ret*+/+ cell (green arrow) moves to the right, towards a tip, passing a red *Ret*−/− cell (red arrow). **D**, fragmentation of a dying *Ret*−/− cell (arrows). Scale bars, 50 μm.
(TIF)

S5 Fig Distribution of WT/mutant cell ratios among *Ret*-MADM clones in E17.5 kidneys. The histogram shows the number of clones with WT/mutant cell ratios in the indicated ranges. Orange bars indicate clones with more mutant than WT cells, the grey bar indicates clones with equal numbers of WT and mutant cells, and blue bars indicate clones with more WT than mutant cells. The numbers below each bar indicate the range of WT/mutant cell ratios for that category, and the average number of cells per clone in each category. Note that the clones with a high ratio of WT/mutant cells tend to be very large clones, while those with more similar numbers of WT and mutant cells tend to be smaller clones. Data available from the Dryad Digital Repository: http://dx.doi.org/10.5061/dryad.pk16b [42].
(TIF)

S1 Movie The movie includes the complete time lapse series corresponding to Fig 1A–1D. (MP4)

S2 Movie Lineage tracing of *Ret*-expressing ureteric bud tip cells in renal organ culture. A *Ret-CreERT2/+*; *Rosa26R-tdTomato/+*; *Hoxb7-myrVenus/+* kidney was excised at E12.5, treated for 1 h with 1 μm 4-OH tamoxifen, washed several times with PBS and cultured in normal medium. Images were collected every 20 min. The image on the left shows the green channel (showing the entire ureteric bud epithelium) and the red channel (showing tdTomato+ cells), while the image on the right shows only the red channel. At ~10 h, red cells start to appear in the extreme tips of the ureteric bud. As the tips extend and branch, the tips remain tdTomato+, as do the newly formed trunks (indicated by arrows in last frame). The yellow numbers at bottom left show the elapsed time since the start of the movie (h:min:s on day 1 and d:h:min:s on days 2–3); there is a gap in the movie between 21h:22 min and 1d:1h:9min.
(MP4)

S3 Movie The movie includes the complete time lapse series corresponding to Fig 2A–2F. (MP4)

S4 Movie The movie includes the complete time lapse series corresponding to Fig 4A–4G. (MP4)

S5 Movie The movie includes the complete time lapse series corresponding to Fig 6G–6I. (MP4)

## Abbreviations

BMP: bone morphogenetic protein
ETS: E26 transformation-specific
Etv5^M^: *Etv5* ^*tm1Kmm*^
FGF: fibroblast growth factor
MACD: mitosis-associated cell dispersal
MADM: Mosaic Analysis with Double Markers
MADM6: MADM sequence elements on chromosome 6
MADM11: MADM sequence elements on chromosome 11
UB: ureteric bud
