# Supplementary figures and images for: Ret and Etv4 Promote Directed Movements of Progenitor Cells during Renal Branching Morphogenesis
Source: PLoS Biol. 2016 Feb 19;14(2):e1002382. doi: 10.1371/journal.pbio.1002382 (PMC4760680; doi:10.1371/journal.pbio.1002382)

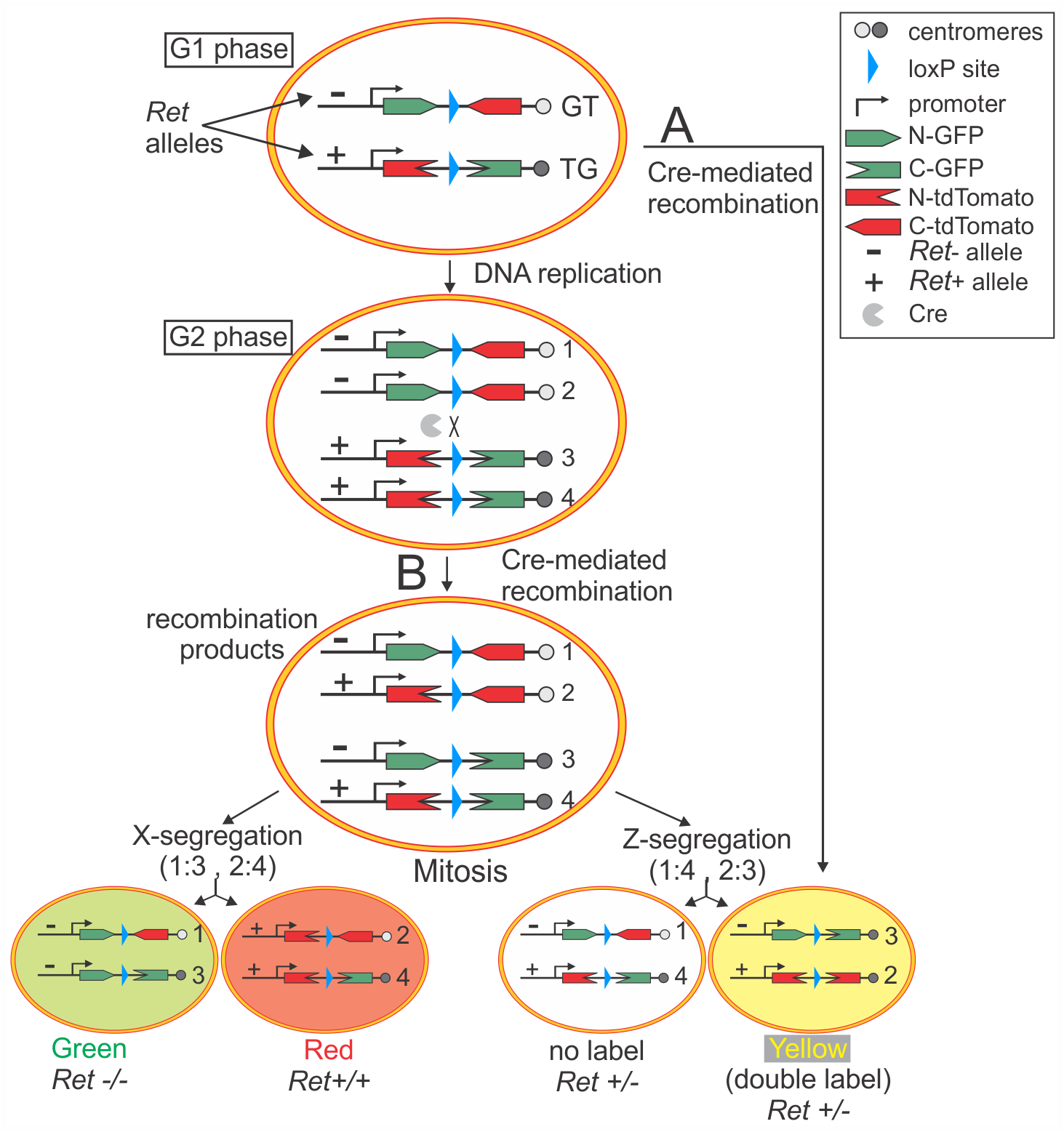

Supplement: S1 Fig — The key in the upper right explains the symbols used. In the starting cell (top diagram), shown in G1 phase, one chromosome 6 homolog carries the MADM-GT allele [34,36] at the Rosa26 locus in cis to a Ret-null allele (−), and the other chromosome 6 homolog carries the MADM-TG allele at the Rosa26 locus in cis to a wild-type Ret allele (+). Thus, the cell is heterozygous for Ret. Neither the MADM-GT nor MADM-TG allele expresses a functional fluorescent protein [34,36]. If Cre-mediated recombination occurs during G1 phase (or during G0, or in postmitotic cells), it generates a functional Tomato (red fluorescent protein) gene and a functional GFP gene in the same diploid cell, so the cell is double-labeled and appears yellow. It also remains heterozygous for Ret. If Cre-mediated recombination occurs during G2, after DNA replication, the recombination products include a functional Tomato gene in cis to a Ret wild-type allele, and a functional GFP gene in cis to a Ret-null allele (shown on chromatids 2 and 3, respectively), as well as two non-recombined, non-functional MADM alleles (on chromatids 1 and 4). At mitosis, the four chromatids segregate in either of two patterns, X-segregation or Z-segregation. The former yields a Ret−/−, GFP-expressing cell and a Ret +/+, Tomato-expressing cell. The latter yields an unlabeled cell and a double-labeled (yellow) cell, both of which remain Ret+/−. Subsequent cell divisions preserve the genotype and fluorescent protein expression of the initial recombinant cells. The strategy for Etv4-MADM was similar, except we used MADM alleles on chromosome 11 [37], where Etv4 is located. Diagram modified from [34]. (TIF) [file pbio.1002382.s001.tif]

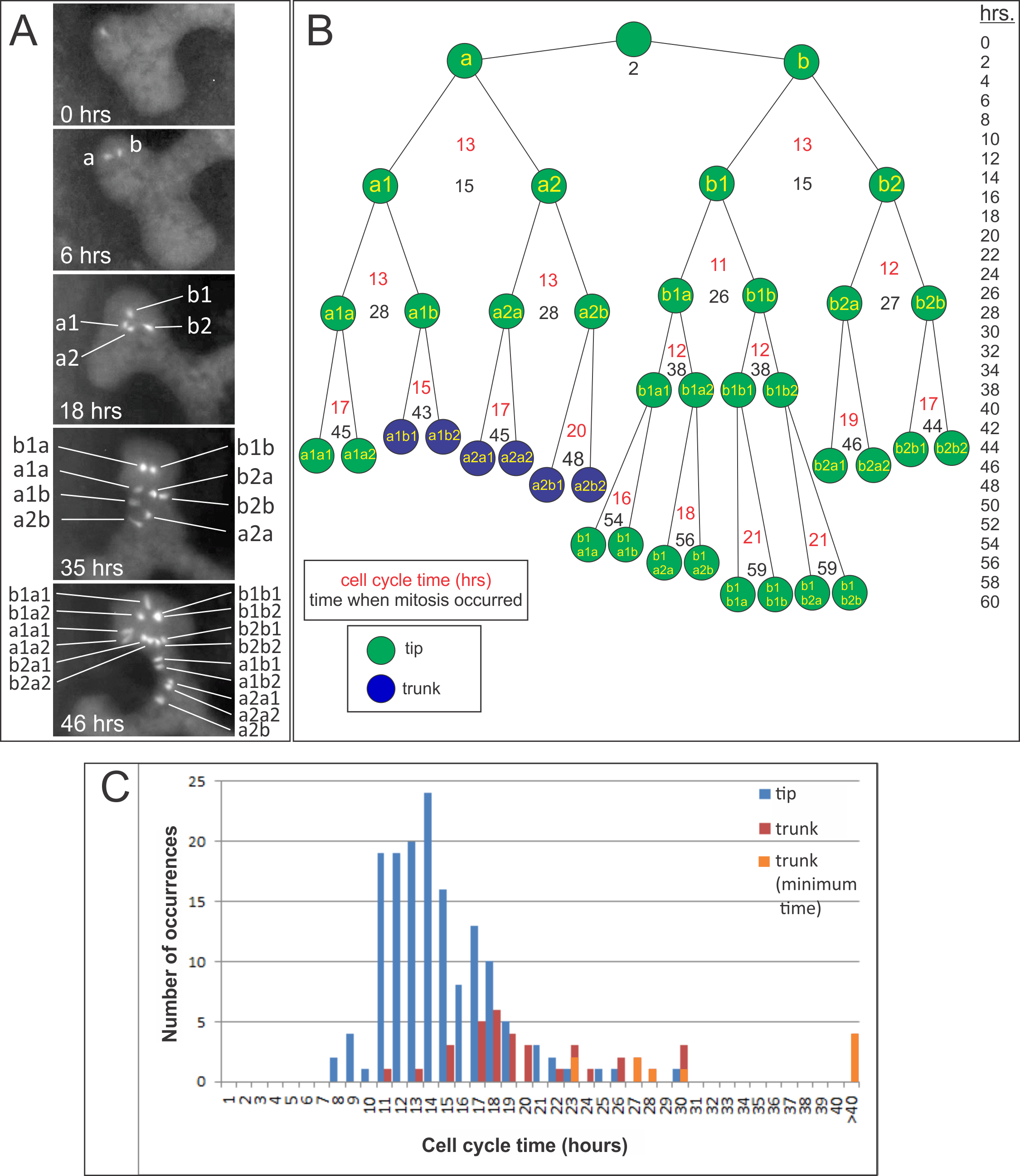

Supplement: S2 Fig — Cell cycle times were measured by noting when each labeled cell divided. A, images of a cultured kidney (the same one shown in Fig 1A), in which the identity of each cell in a labeled clone is marked. B, the complete lineage of the clone shown in A, from 0 to 59 h. The y-axis indicates the time of cell division (also shown in black numbers between each pair of sister cells). Red numbers indicate the time between two successive mitoses, i.e., cell cycle time. Green circles indicate cells that divided in the tip, and blue circles indicate cells that divided in the trunk. C, distribution of cell cycle times in the tip and trunk, based on 150 intermitotic intervals in UB tips and 33 in UB trunks. For trunk cells, red bars indicate the time between two successive mitoses, and orange bars indicate the time from a cell division until the end of the movie (i.e., the minimum cell cycle time). Cells were classified as “tip” or “trunk” based on their location at the end of the cell cycle (i.e., the second of the two successive mitoses) or at the end of the movie, whichever came first. Data available from the Dryad Digital Repository: http://dx.doi.org/10.5061/dryad.pk16b [42]. (TIF) [file pbio.1002382.s002.tif]

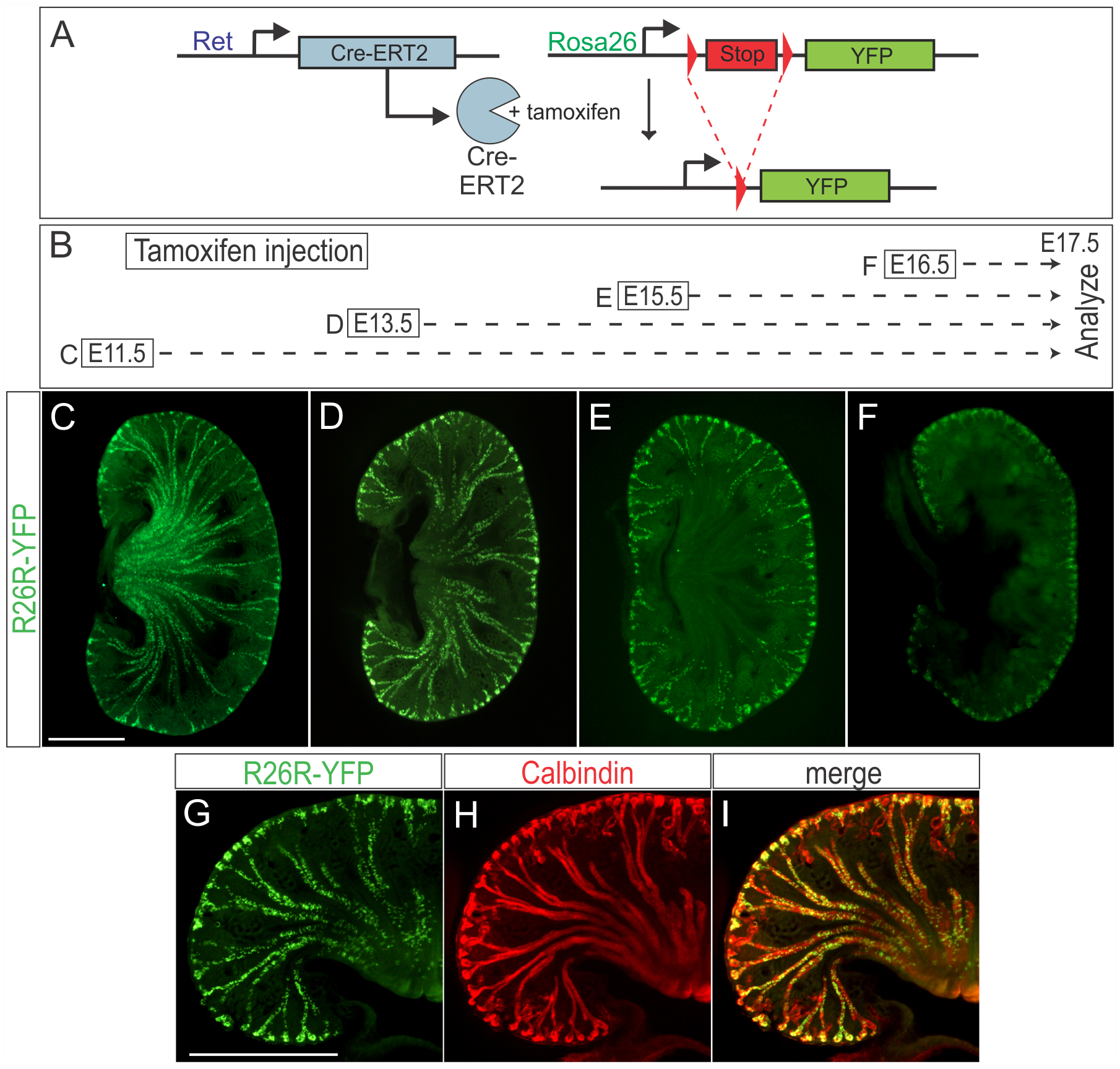

Supplement: S3 Fig — A, genetic strategy for fate-mapping Ret-expressing cells. To follow the fate of UB tip cells at different stages of kidney development in vivo, we used a transgenic line, Ret CreERT2, in which a tamoxifen-inducible form of Cre was targeted to the Ret locus and is thus expressed in the pattern of the Ret gene [77]. Ret CreERT2 mice were crossed with Rosa26R YFP mice, in which YFP is permanently expressed from the Rosa26 locus only after a floxed “stop” sequence is removed by Cre-mediated recombination [47,81]. B, timing of tamoxifen injection and analysis. Pregnant females were injected with a single 2 mg dose of tamoxifen at E11.5, E13.5, E15.5, or E16.5. This induces Cre activity starting about 6–8 hours later, and continuing for about 24 hours [82,83]. The embryos were all dissected at E17.5, the kidneys were vibratome-sectioned (50 μm), and YFP fluorescence was photographed. As expected, given the tip-restricted expression of Ret in the UB throughout kidney development [12] (GUDMAP.org), when recombination was induced at E16.5, YFP was expressed at E17.5 only in cells close to the UB tips, at the edge of the kidney (F). In contrast, when recombination was induced at E11.5 (when Ret is expressed broadly in the first two UB branches), YFP+ cells were found at E17.5 all along the collecting ducts, from the papilla to the distal tips (C). When recombination was induced at E13.5, YFP+ cells were found at E17.5 throughout most of the collecting ducts, except for the papillary region (D); and when it was induced at E15.5, YFP+ cells were found at E17.5 from the cortical CDs to the tips, but not in the medullary or papillary regions (E). As expected, all cells labeled by Ret CreERT2 remained within the collecting ducts, as confirmed by costaining for YFP and calbindin, a collecting duct marker (G-I). Scale bars: 500 μm. (TIF) [file pbio.1002382.s003.tif]

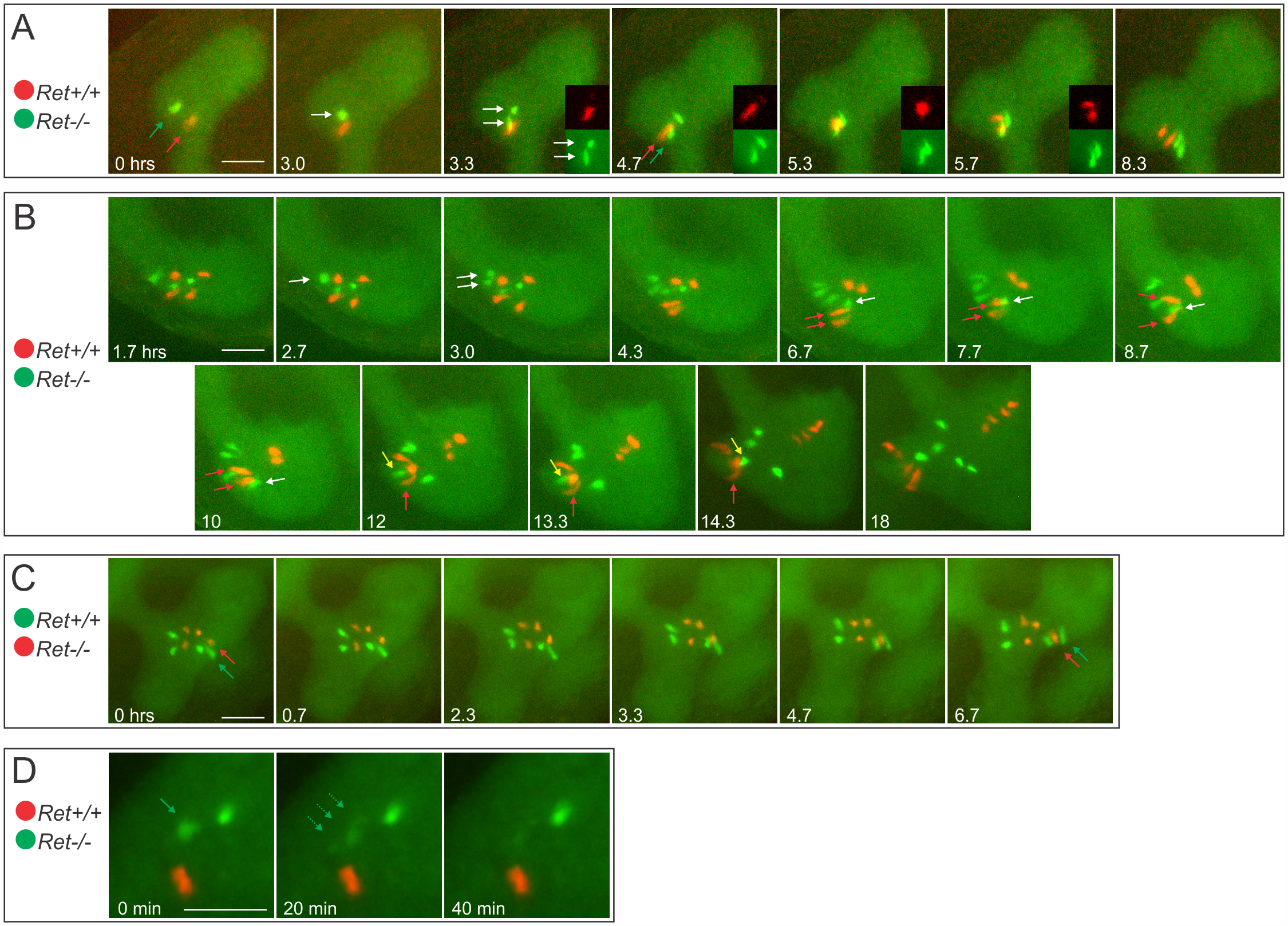

Supplement: S4 Fig — A–C, three examples of MACD and cell rearrangement in Ret-MADM clones (B shows the same clone as Fig 3B, and C shows the same clone as Fig 3F, but additional time points are shown here to highlight MACD and other cell movements). A, the green Ret−/− cell (green arrow) starts closer to the left UB tip than the red Ret+/+ cell (red arrow), but by 4.7 h they have exchanged positions. The Ret−/− cell divides between 3 and 3.3 h (white arrows) and the Ret+/+ cell divides between 5.3 and 5.7 h, both displaying MACD (seen as rounding of the parental mitotic cell, and in the next frame as two non-contiguous daughter cells). Between 5.7 and 8.3 h, both Ret+/+ cells continue to move closer to the left UB tip than the two Ret−/− cells. The insets show the separate red and green channels when a red and green cell overlap, between 3.3 and 5.7 h. B, a green Ret−/− cell (white arrow) undergoes MACD between 2.7 and 3.0 h. Between 6.7 and 10 h, two red Ret+/+ cells (red arrows) move past a green Ret−/− cell (white arrow) towards the site where a new tip is forming. Between 12 and 14.3 hrs, three red Ret+/+ cells (red arrow) move left towards the site of tip outgrowth, passing a green Ret−/− cell (yellow arrow). C, in a clone at the center of a T-shaped branching tip, a green Ret+/+ cell (green arrow) moves to the right, towards a tip, passing a red Ret−/− cell (red arrow). D, fragmentation of a dying Ret−/− cell (arrows). Scale bars, 50 μm. (TIF) [file pbio.1002382.s004.tif]

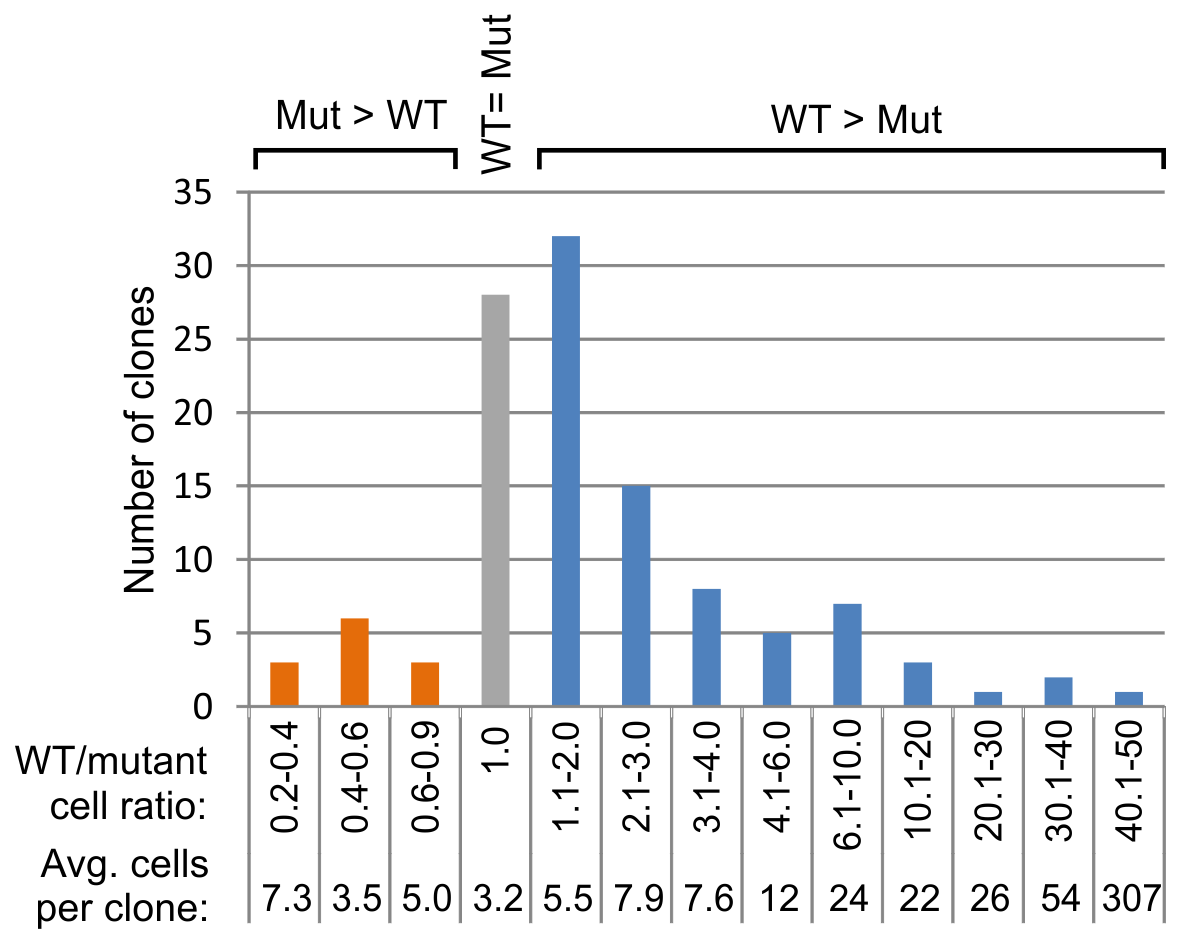

Supplement: S5 Fig — The histogram shows the number of clones with WT/mutant cell ratios in the indicated ranges. Orange bars indicate clones with more mutant than WT cells, the grey bar indicates clones with equal numbers of WT and mutant cells, and blue bars indicate clones with more WT than mutant cells. The numbers below each bar indicate the range of WT/mutant cell ratios for that category, and the average number of cells per clone in each category. Note that the clones with a high ratio of WT/mutant cells tend to be very large clones, while those with more similar numbers of WT and mutant cells tend to be smaller clones. Data available from the Dryad Digital Repository: http://dx.doi.org/10.5061/dryad.pk16b [42]. (TIF) [file pbio.1002382.s005.tif]
